# Living with marginal coral communities: Diversity and host-specificity in coral-associated barnacles in the northern coral distribution limit of the East China Sea

**DOI:** 10.1371/journal.pone.0196309

**Published:** 2018-05-01

**Authors:** Benny K. K. Chan, Guang Xu, Hyun Kyong Kim, Jin-Ho Park, Won Kim

**Affiliations:** 1 Biodiversity Research Center, Academia Sinica, Taipei, Taiwan; 2 School of Biological Sciences, Seoul National University, Seoul, Korea; National Taiwan Ocean University, TAIWAN

## Abstract

Corals and their associated fauna are extremely diverse in tropical waters and form major reefs. In the high-latitude temperate zone, corals living near their distribution limit are considered marginal communities because they are particularly extremely sensitive to environmental and climatic changes. In this study, we examined the diversity and host usage of coral-associated barnacles on Jeju Island, Korea, the northern coral distribution limit in the East China Sea. In this study, only three coral-associated barnacles—from two genera in two subfamilies—were collected. The Pyrgomatinid barnacles *Cantellius arcuatus* and *Cantellius* cf. *euspinulosum* were found only on the corals *Montipora millepora* and *Alveopora japonica*, respectively. The Megatrematinid barnacle *Pyrgomina oulastreae*, relatively a generalist, was found on *Psammocora* spp. (both *profundacella* and *albopicta*) and *Oulastrea crispata* corals. The host usage of these three barnacles does not overlap. DNA barcode sequences of the *C*. *arcuatus* specimens collected in the present study matched those collected in Kochi in Japan, Taiwan, Malaysia and Papua New Guinea, suggesting that this species has a wide geographical distribution. *C*. *arcuatus* covers a wider host range in Taiwan waters, inhabiting *Montipora* spp. and *Porites* spp., which suggests that the host specificity of coral-associated barnacles varies with host availability. *C*. cf. *euspinulosum* probably has a very narrow distribution and host usage. The sequences of *C*. cf. *euspinulosum* on Jeju Island do not match those of any known sequences of *Cantellius* barnacles in the Indo-Pacific region. *P*. *oulastreae* probably prefers cold water because it has been reported in temperate regions. Coral-associated barnacles in marginal communities have considerably lower diversity than their subtropical and tropical counterparts. When host availability is limited, marginal coral-associated barnacles exhibit higher host specificity than those in subtropical and tropical reef systems.

## Introduction

Coral reef ecosystem supports a high diversity of scleractinian corals and their associated fauna. More than 56% of coral-associated fauna form obligate symbiotic relationships with their coral hosts [1], and these associated fauna are major contributors to the overall biodiversity of the coral system [2]. The diversity of corals and their associated fauna is particularly high in the Coral Triangle region, which is considered a marine biodiversity hotspots. The distribution of coral reefs is limited by water temperature because hermatypic corals cannot survive in an environment with winter temperatures below 14–18°C [3]. At high latitudes, where the water temperature is approximately 14°C, corals living at the limit of their geographical distribution are mainly composed of encrusting forms and exhibit considerably reduced growth and reproduction. Consequently, these high-latitude corals do not form intense reefs, and are called marginal communities. Studying the basic ecology of corals and their associated fauna is particularly crucial for conserving marginal coral reefs because they are very sensitive to impending environmental changes [4]. Species diversity of corals at high latitudes and in marginal communities has been studied in some locations [5, 6]. However, the diversity of coral-associated fauna and their host usage in marginal communities have received limited attention.

Coral-associated barnacles are among the common obligate symbionts of scleractinian corals, and most species are classified in the family Pyrgomatidae under the order Sessilia [7]. The bases of coral-associated barnacles are cup shaped and embedded in the skeleton of their host corals. The external calcified shells are overgrowths by the coral tissues. Coral-associated barnacles are principally suspension feeders but ^13^C stable isotope studies have shown that the organic matter produced by coral zooxanthellae contributes to some carbon in the barnacles [8, 9]. In return, ammonium released from the coral-associated barnacles is absorbed by the zooxanthellae in the corals. To date, >70 species of coral-associated barnacles have been reported worldwide, with variable degrees of host specificity [10–13].

In the West Pacific region, studies on the species diversity of coral-associated barnacles have been focused on tropical and subtropical coral reefs, where coral diversity is extremely high (e.g., Hong Kong: [14]; Taiwan: [10, 11, 15]; the Philippines: [16]). More than 50 coral-associated barnacle species have been recorded in the tropical and subtropical waters of the West Pacific, including a considerable number of specialists and generalists with respect to their coral hosts [7]. Coral-associated barnacles in high-latitude regions probably exhibit different assemblages or host ranges. Most studies on barnacles associated with high-latitude corals have been conducted on the Pacific coast of Japan, under the influence of the warm Kuroshio Current. In this region, 30 coral-associated barnacle species from 100 species of coral hosts have been recorded [17].

Jeju Island is located in the southwestern waters of Korea, in the East China Sea, and the hydrology is influenced by the warm Tsushima Current, a branch of the Kuroshio Current, as well as the Yellow Sea runoff. Because of the low seawater temperature in winter and the minor influence from the Kuroshio Current, the island’s benthic communities are mainly dominated by macroalgae and soft corals. Only eight species of zooxanthellate scleractinian corals and a few species of nonzooxanthellate corals have been recorded on Jeju Island [6, 18]. The waters near Jeju Island are considered one of the northern limits of coral distribution in the East China Sea [6]. However, the diversity and host range of coral-associated barnacles at the northern limit of coral distribution are relatively unknown compared with that of barnacles on high-latitude corals in the Pacific Ocean. Only two species of coral-associated barnacles have been reported thus far on Jeju Island [19]. The objective of the present study was to examine the diversity of coral-associated barnacles and their host relationship on Jeju Island, Korea.

## Materials and methods

### Ethics statement

Permission to collection was granted by the Jeju Special Self-Governing Province (No. 2436, 2016).

### Study sites and timing

**Fig 1:**
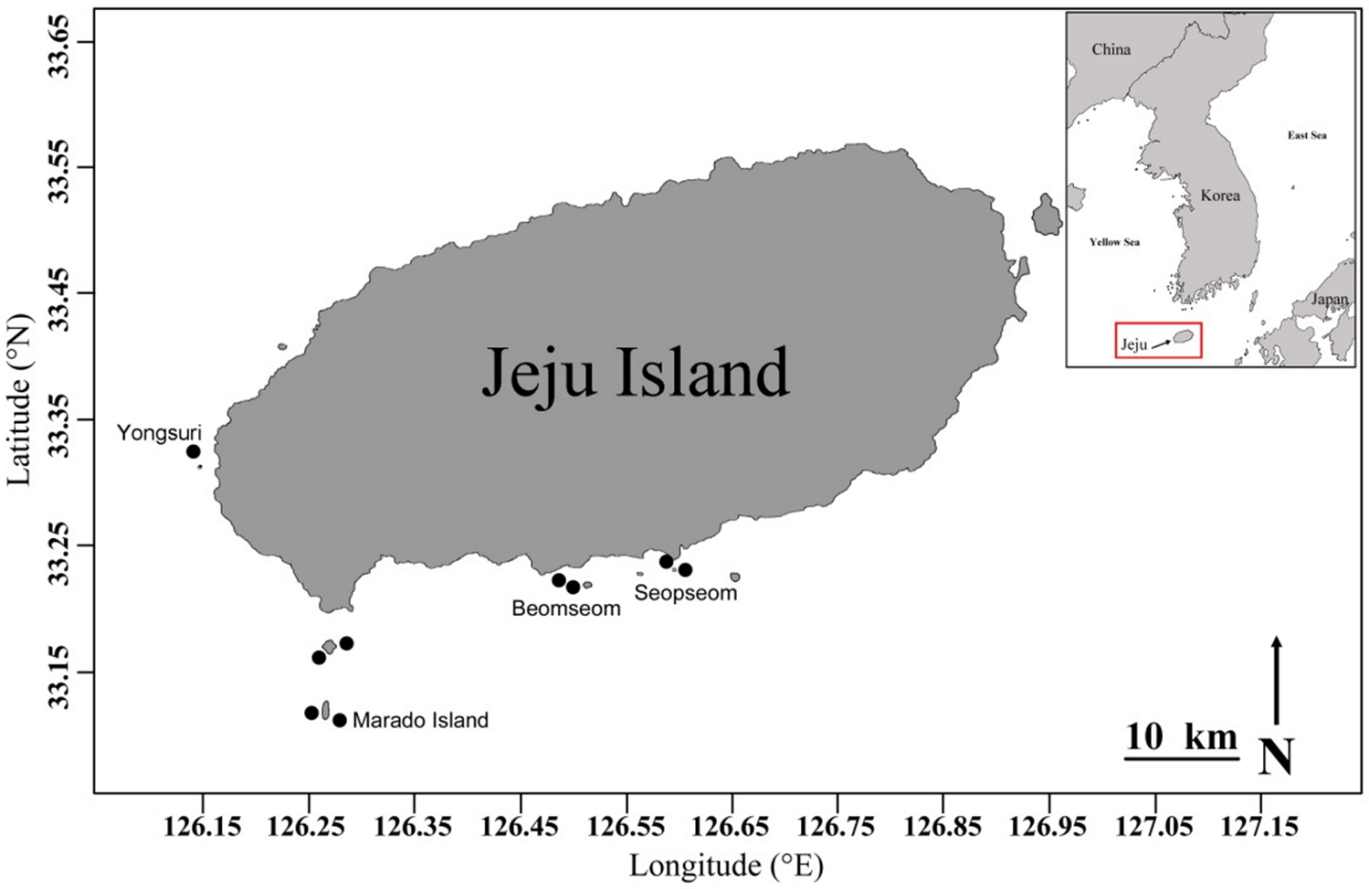
Map of Jeju Island, Korea. Showing the collection sites of coral-associated barnacles used in this study.

In total, nine sites were selected in the southern waters of Jeju Island in August 2016 to sample coral-associated barnacles (Fig 1). Coral-associated barnacles were sampled through scuba diving at depths of 5–20 m. Before sampling the coral-associated barnacles, the entire piece of coral with the barnacles was photographed *in situ* for coral identification. Small pieces of coral with embedded barnacles (approximately 5 × 5 cm) were collected using hammer and chisel at a 5–20 m depth through scuba diving. All barnacles and host corals were preserved in 95% EtOH.

### Relative coral abundance

The coral diversity and abundance at the collection site were quantified using the point transect techniques at Seop Seom (northeast [NE] and southeast waters) and Beom Seom, where coral abundance is relatively higher than that at other sites. At each site, 30-m transects were established at 5–20 m. Coral species in every 1-m interval of the transect were photographed and identified. The percentage abundance of coral species at each site was calculated as the cumulative point abundance divided by the total point abundance (i.e. 30 m = 30). Coral species were identified using the key and guides by Sugihara *et al*. [6].

### Abundance of barnacles on corals

For each barnacle specimen collected, the entire piece of coral with the barnacles was photographed *in situ* with a 9 cm scale bar before collection. Number of coral-associated barnacles on each piece of coral hosts was measured from an equivalent 9 cm x 9 cm quadrat = 81 cm^2^ area inside each digital photograph. Variation in barnacle abundance on each coral host (number per 81 cm^2^ quadrat) was analysed using One-Way Analysis of Variance (ANOVA).

### Morphological examination

Barnacles were isolated from the host corals using forceps, and the morphological characteristics of shell parts (shells, scutum and tergum) and somatic bodies (six pairs of cirri, penis, and oral cone) were examined. Organic debris and coral tissue on the surface of the shells, scutum and tergum were removed using forceps and further cleaned ultrasonically (for 2–5 s). The cleaned shells and opercular valves (scutum and tergum) were immersed in 1.5% bleach for approximately 5 h to completely digest the organic tissue, and the shells were then rinsed with slow-running purified water for 30 min and air-dried. The shells, scutum and tergum were gold coated and observed under SEM, following the methods of Chan *et al*. [11]. The cirri, penis and oral cone were dissected from the somatic bodies and examined using a light microscope (Zeiss Scope A1, Zeiss, Germany) with high definition lenses (Zeiss Plan APO Chromat 40X/0.95 and ZEISS Plan APO Chromat 100x/1.4 oil), which allowed clear observation of setal types on the cirri and mouth parts. The setal descriptions follow those of Chan *et al*. [20]. The rostral–carinal basal diameter of the collected specimens was measured using a digital caliper (±0.1 mm). All barnacle specimens were housed in the barnacle collection of the Coastal Ecology Laboratory of Academia Sinica, Taiwan.

### Molecular analysis

Total genomic DNA was extracted from the soft tissue of barnacle specimens using the Qiagen QIAquick Tissue Kit (Chatsworth, CA, USA) following the manufacturer’s instructions. Partial sequences of the mitochondrial genes 12S rDNA (12S) and cytochrome c oxidase subunit I (COI) were amplified using polymerase chain reaction with the primers 12S-FB and 12S-R2 [12] and COI-F5 5′ AAACCTATAGCCTTCAAAGCT 3′ and COI-R4 5′ GTATCHACRTCYATWCCTACHG 3′ [21], respectively. Mitochondrial markers including COI and 12S are useful for species delineations in coral-associated barnacles and a number of studies have used these two markers for new species descriptions [22–24]. There are large numbers of mitochondrial gene sequences from coral-associated barnacles available from the Genbank. The use of mitochondrial markers in the present study makes it possible to compare species diversity data from Jeju waters with other available mitochondrial sequences. The PCR solution contained 40 ng of template DNA, 5 μL of Taq DNA Polymerase Master Mix (1.5 mM MgCl_2_; Ampliqon, Denmark), 1 μM of each primer, and ddH_2_O for a total volume of 10 μL. PCR was conducted under the following conditions: 2 min at 95°C for initial denaturation, 35 cycles of 30 s at 95°C, 1 min at 48°C, 1 min at 72°C, and a final extension for 5 min at 72°C. The PCR products were then purified using a DNA gel purification kit (Tri-I Biotech, Taipei, Taiwan). Direct sequencing of the purified PCR products was performed using the ABI 3730XL Genetic Analyzer with BigDye terminator cycle sequencing reagents (Applied Biosystems, Foster City, CA, USA).

DNA sequences were proofread using MEGA v. 7 [25] and aligned with the *Cantellius* sequences from GenBank through multiple alignment using MAFFT v. 6.717 [26]. Alignments were also examined visually and ambiguous positions were adjusted manually. A matrix of genetic distances within and among the species was generated using Kimura’s two-parameter model in MEGA v. 7. The stability of clades was evaluated using bootstrap tests with 1,000 replications. A maximum likelihood (ML) test was conducted for concatenated datasets (mitochondrial COI + 12S). ML analysis was performed using RAxML-HPC2 on XSEDE [27] through the online server Cyberinfrastructure for Phylogenetic Research (CIPRES) with the GTRGAMMA model of nucleotide substitution and 1,000 bootstrap replicates. For analysis, other *Cantellius* and pyrgomatid species available from the Genbank were used for comparisons (S1 Table) and *Amphibalanus amphitrite* was selected as an outgroup. The use of *A*. *amphitrite* as an outgroup candidate is appropriate for molecular phylogenetic analysis of coral-associated barnacles because from a previous study on molecular phylogeny of coral-associated barnacles [13], the coral-associated barnacle clade (pyrgomatid clade) is sister to balanid clade including *A*. *amphitrite*.

## Results

### Coral abundance

**Fig 2:**
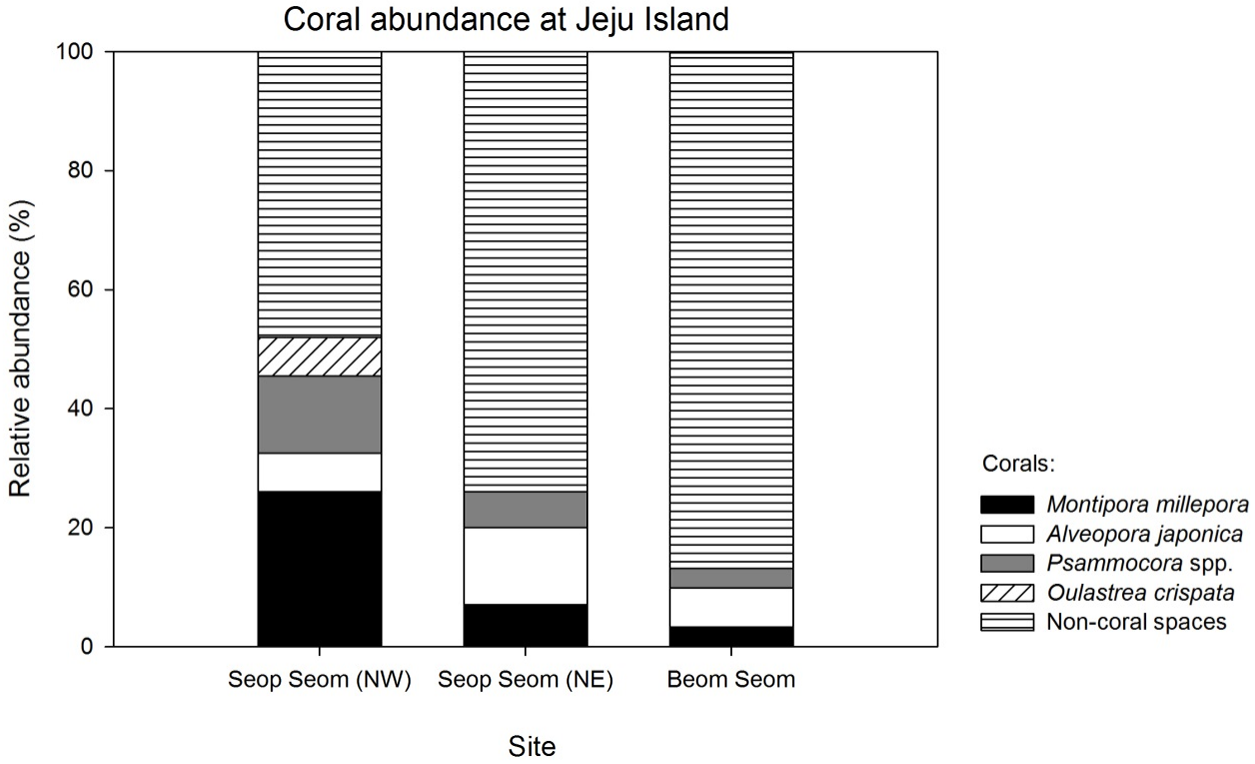
Relative abundance of scleractinian corals in south and west waters of Seop Seom and Beom Seom off of Jeju waters from 30 metres transect surveys. *Psammocora* spp. include both *P*. *profundacella* and *P*. *albopicta*, which are difficult to identify *in situ* in the field without examining the columellae structure from the coral skeleton. Non-coral surfaces were colonized by soft corals, kelps and sponges.

The total coral abundance was 52% in northwest (NW) Seop Seom, 22% in NE Seop Seom, and approximately 10% in Beom Seom. Five species of corals were sampled, namely *Montipora millepora*, *Psammocora profundacella*, *P*. *albopicta*, *Oulastrea crispata* and *Alveopora japonica*. In NW Seop Seom, *M*. *millepora* had the highest relative abundance, with 26% coverage. *A*. *japonica* was more abundant in NE Seop Seom. *Psammocora* spp. and *O*. *crispata* were relatively less abundant among the sites studied (Fig 2).

### Barnacle diversity, abundance and host usage

**Fig 3:**
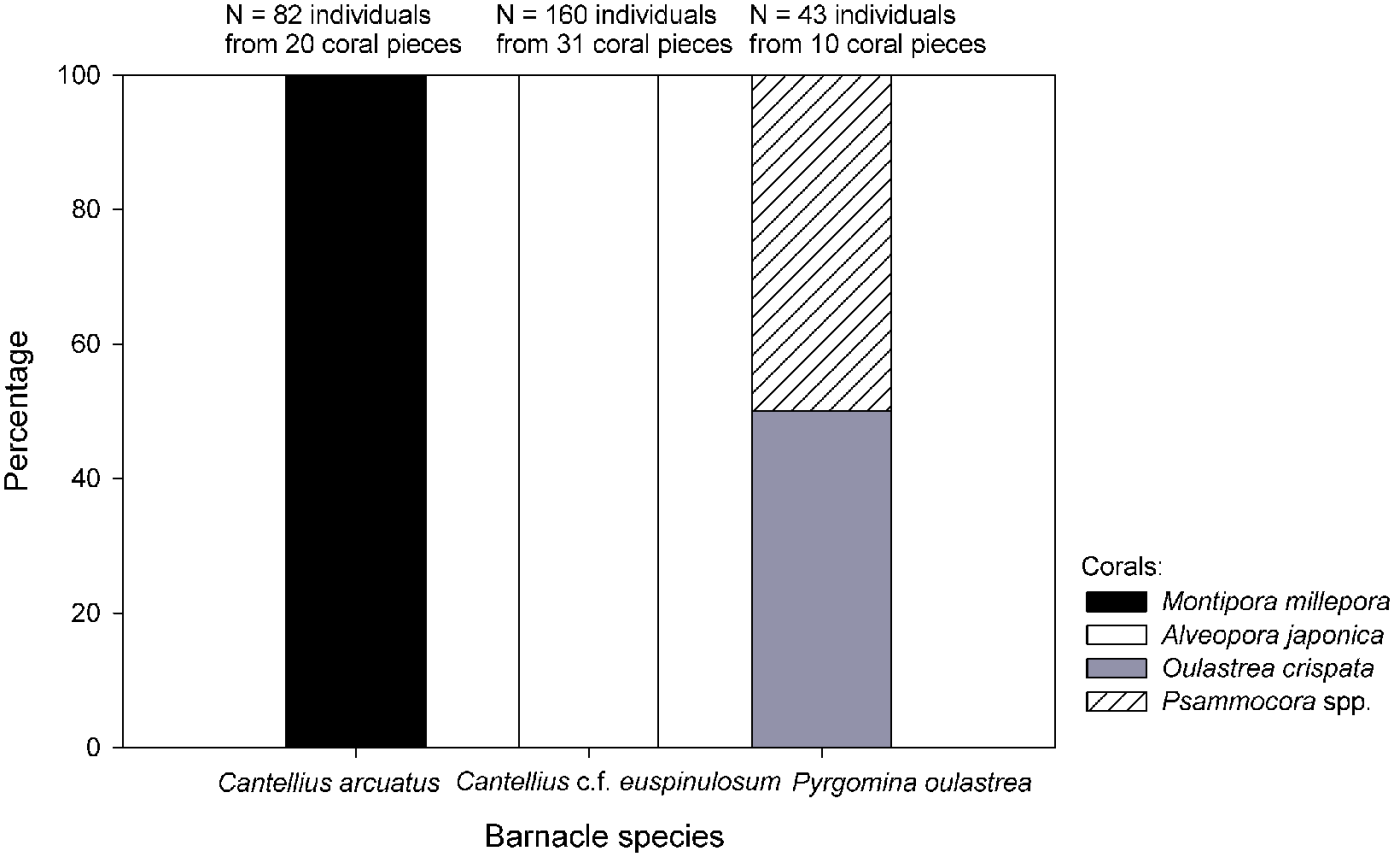
Coral host usage by barnacles *Cantellius arcuatus*, *C*. cf. *euspinulosum* and *Pyrgomina oulastreae* in Jeju Island. Note *C*. *arcuatus* and *C*. cf. *euspinulosum* were 100% found on the coral *Montipora millepora* and *Alveopora japonica* respectively. *Pyrgomina oulastreae* are found on both *Psammocora* spp. and *Osulastrea crispata*. *Psammocora* spp. include both *P*. *profundacella* and *P*. *albopicta*.

Among all the sites sampled, three species of barnacle covering two genera from two subfamilies were identified, namely the Pyrgomatinids *Cantellius arcuatus* and *Cantellius* cf. *euspinulosum* and the Megatrematinid *Pyrgomina oulastreae* (see S1 File for detailed taxonomic description of barnacle species). Among the eight sites studied, *C*. *arcuatus* was found exclusively on the coral *M*. *millepora*. *C*. cf. *euspinulosum* was exclusively present on *A*. *japonica*. *P*. *oulastreae* was present on a number of coral hosts, such as *Psammocora* spp. (both *profudacella* and *albopicta*) and *Oulastrea crispata*, but it was absent from *Montipora* and *Alveopora* (Fig 3).

**Fig 4:**
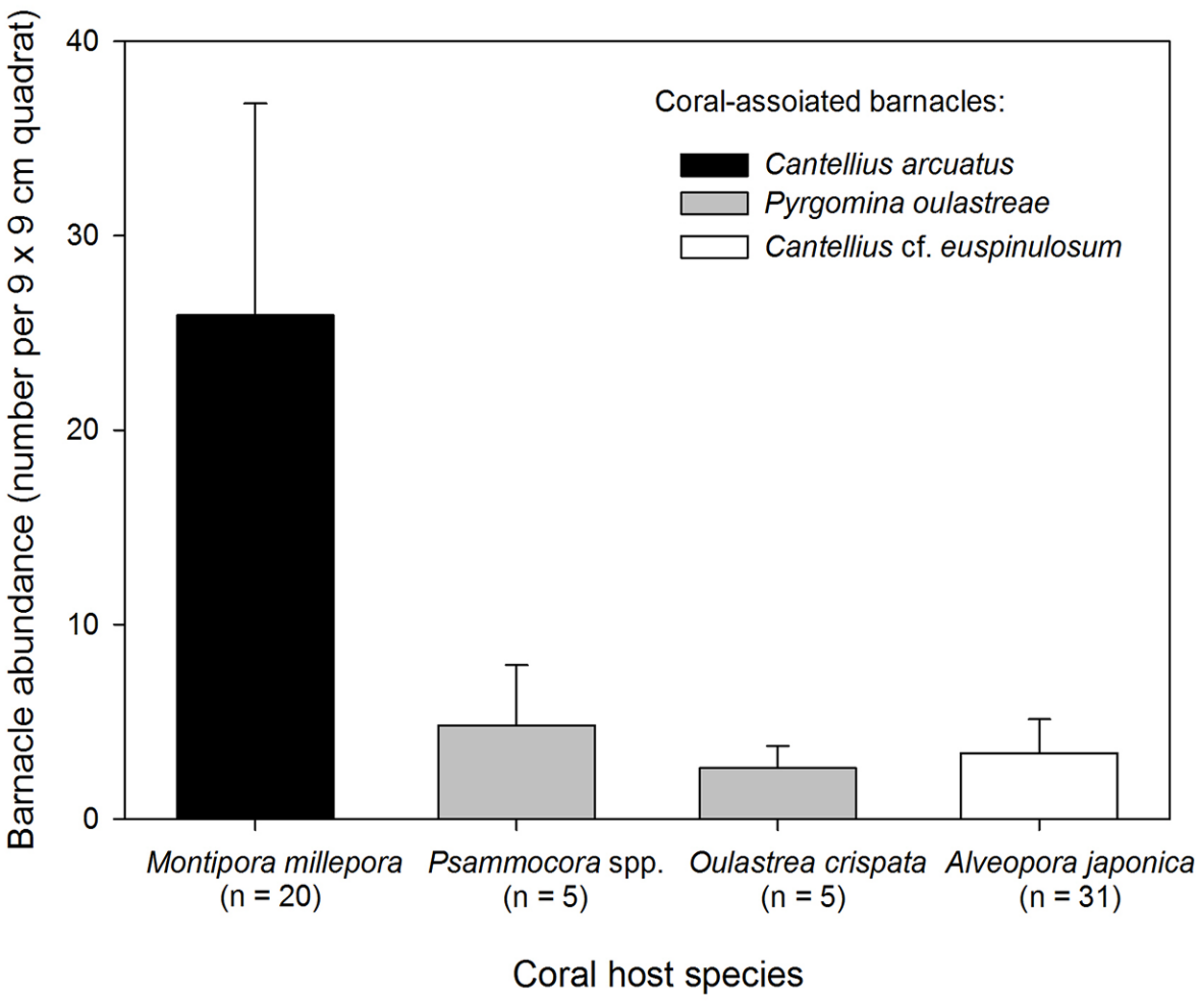
Mean abundance (+1 standard deviation) of coral-associated barnacles *Cantellius arcuatus*, *C*. cf. *euspinulosum* and *Pyrgomina oulastreae* on their coral hosts *Montipora millepora*, *Psammocora* spp., *Osulastrea crispata* and *Alveopora japonica*, respectively, in Jeju Island. *Psammocora* spp. include both *P*. *profundacella* and *P*. *albopicta*.

*Cantellius arcuatus* has the highest abundance on its coral host *Montipora millepora*, reaching an average of 25 individuals per 81 cm^2^ (Fig 4). Compared to *C*. *arcuatus*, abundance of *Pyrgomina oulastreae* on *Psammocora* and *Oulastrea* corals are lower, reaching an average about 4.8 and 2.6 individuals per 81 cm^2^ respectively (Fig 4). *Cantellius* cf. *euspinulosum* has an average of 3.4 individuals per 81 cm^2^. One-Way ANOVA showed that barnacle abundances on different corals were significant, F _(3, 57)_ = 82.84, p < 0.05. Pairwise SNK results showed that *Cantellius arcuatus* on the coral *M*. *millepora* has significantly higher abundance than *P*. *oulastreae* on *Psammocora* and *Oulastrea* corals and *C*. cf. *euspinulosum* on *Alveopora* coral, whilst *P*. *oulastreae* and *C*. cf. *euspinulosum* have similar abundances on their coral hosts (Fig 4).

### Molecular analysis of coral-associated barnacles

**Fig 5:**
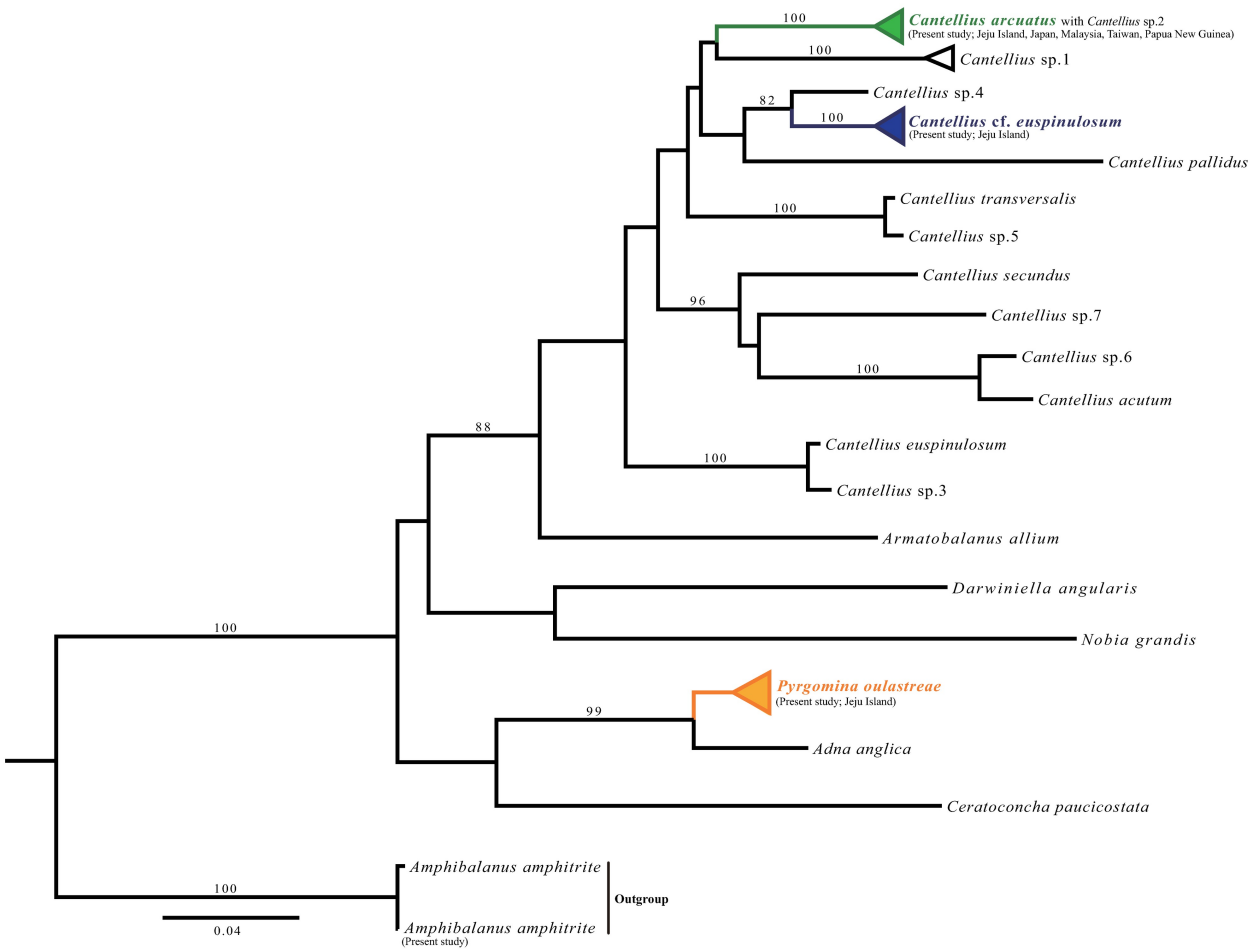
Phylogenetic tree for coral-associated barnacles based on maximum likelihood analysis of the two mitochondrial gene datasets, using *A*. *amphitrite* as outgroup. Bootstrap scores > 80 are presented at the major nodes. The scale bar indicates the number of substitutions per site.

Phylogenetic trees from COI and 12S revealed similar patterns, except the species *Adna anglica* and *Pyrgomina oulastreaea* were not differentiated in the 12S marker (S2 File). The multiple sequence alignment revealed that the K2P distance among the sequences ranged from 0.2% to 20.5% (Table 1). The intraspecific divergence of *C*. *arcuatus* was 0.4% (Table 1), and it was clustered in the same clade as the sequence of *Cantellius* sp. 2 (12S, HG970552 and CO1, HG970494) and with *C*. *arcuatus* collected from Malaysia, Kochi in Japan, Taiwan and Papua New Guinea (Fig 5) in the concatenated tree (COI+12S). From the concatenated tree, *C*. cf. *euspinulosum* collected in the present study was clustered in its own clade, and it differed from *C*. *euspinulosum* collected in Taiwan (Fig 5). Furthermore, their distant relationship was supported by the K2P distance, which was 9.1% (Table 1). *P*. *oulastreae* was clustered into its own clade, and located in another major clade containing *Adna* and *Ceratochoncha* (Fig 5).

**Table 1:** Mean K2P distance for concatenated dataset between and within the studied species.

**Species**	**1**	**2**	**3**	**4**	**5**	**6**	**7**	**8**	**9**	**10**	**11**	**12**	**13**	**14**	**15**	**16**	**17**	**18**	**19**	**20**	**21**
1. *Cantellius acutum*	-																				
2. *Cantellius arcuatus*	0.088	0.004																			
3. *Cantellius* cf. *euspinulosum*	0.105	0.074	0.005																		
4. *Cantellius euspinulosum*	0.099	0.084	0.091	-																	
5. *Cantellius pallidus*	0.103	0.117	0.105	0.109	-																
6. *Cantellius* sp. 1	0.104	0.091	0.093	0.100	0.127	0.014															
7. *Cantellius* sp. 2	0.081	0.003	0.069	0.080	0.107	0.088	-														
8. *Cantellius* sp. 3	0.085	0.080	0.087	0.010	0.103	0.099	0.079	0.039													
9. *Cantellius* sp. 4	0.096	0.074	0.040	0.084	0.091	0.084	0.073	0.086	-												
10. *Cantellius* sp. 5	0.093	0.082	0.088	0.089	0.110	0.103	0.081	0.089	0.084	-											
11. *Cantellius* sp. 6	0.021	0.092	0.098	0.112	0.122	0.117	0.092	0.109	0.100	0.102	-										
12. *Cantellius* sp. 7	0.082	0.100	0.093	0.108	0.119	0.101	0.102	0.111	0.107	0.100	0.091	-									
13. *Cantellius secundus*	0.088	0.095	0.095	0.102	0.118	0.102	0.088	0.094	0.093	0.102	0.096	0.083	-								
14. *Cantellius transversalis*	0.097	0.082	0.093	0.093	0.107	0.101	0.080	0.089	0.082	0.008	0.103	0.097	0.105	-							
15. *Adna anglica*	0.130	0.143	0.137	0.132	0.144	0.129	0.142	0.136	0.141	0.146	0.160	0.148	0.140	0.146	-						
16. *Armatobalanus allium*	0.123	0.116	0.114	0.117	0.139	0.125	0.100	0.100	0.103	0.105	0.119	0.120	0.129	0.120	0.135	-					
17. *Ceratoconcha paucicostata*	0.148	0.140	0.156	0.158	0.149	0.142	0.138	0.164	0.149	0.158	0.172	0.154	0.155	0.156	0.134	0.151	-				
18. *Darwiniella angularis*	0.150	0.148	0.154	0.152	0.169	0.147	0.139	0.142	0.151	0.140	0.141	0.142	0.153	0.158	0.158	0.163	0.163	-			
19. *Pyrgomina oulastreae*	0.145	0.146	0.145	0.139	0.161	0.133	0.144	0.138	0.137	0.152	0.165	0.156	0.139	0.156	0.038	0.141	0.134	0.150	0.007		
20. *Nobia grandis*	0.166	0.171	0.168	0.175	0.172	0.166	0.153	0.165	0.155	0.159	0.166	0.155	0.172	0.175	0.152	0.191	0.161	0.158	0.165	0.006	
21. *Amphibalanus amphitrite*	0.179	0.169	0.166	0.178	0.205	0.171	0.161	0.174	0.166	0.170	0.184	0.176	0.187	0.178	0.165	0.166	0.171	0.170	0.164	0.191	0.002

## Discussion

A total of 17 genera of coral-associated barnacles have been reported in West Pacific waters. In the present study, only three species of coral-associated barnacles—covering two genera and two subfamilies—were identified among five zooxanthellate coral species at Jeju Island. Apparently, the diversity of coral-associated barnacles is affected by interactions between the diversity of coral hosts, latitudinal gradients and oceanographic regimes. From reviewing the diversity of coral-associated barnacles along the latitudinal gradient from Honshu, Shikoku, Kyushu and Okinawa [28], Ogasawara Island [29] and Taiwan [30] and the number of coral species in these regions (Japan: [31], Taiwan: [32]), the diversity of coral-associated barnacle is influenced by both latitudes and coral diversity. At higher latitudes in Boso, Japan, where there are 32 species of corals, only 2 species of coral-associated barnacles were present. In lower latitudes, the Izu and Shikoku regions—which have 80 and 52 coral species respectively—have 8–11 coral-associated barnacle species. Ogasawara Island has 230 corals species and contains 16 coral-associated barnacle species. The highest diversity of coral barnacles in the West Pacific is in Ishigaki, Iriomote Island and Taiwan, each of which has coral species number up to 300, 20–35 coral-associated barnacle species were sampled in these locations.

Oceanographic regimes can in addition affect the diversity of coral-associated barnacles. The Pacific coast of Honshu, Japan (e.g., Wakayama, 33°40′ N and Kochi 32°40′ N) is at latitudes similar to those of the study region in Jeju Island (33°10′ N). However, the high-latitude corals on the Pacific coast of Honshu are influenced by the main warm Kuroshio Current. Up to 70 species of corals [5] and 18 species of coral-associated barnacles [17] have been reported in this region. In Jeju Island, which receives much less influence from the Kuroshio Current, only three species of coral-associated barnacles were collected. In the Japan Sea off Honshu (Hyogo Peninsula, 35°44′ N and Shimane Peninsula, 35°43′ N), only one species, *P*. *oulastreae*, was found [17].

For the three species of coral-associated barnacles collected in the present study, the corals *M*. *millepora* and *A*. *japonica* were more common hosts than *Psammocora* and *Oulastrea*. This, and the finding that *C*. *arcuatus* has a higher abundance than the other two coral-associated barnacle species, suggests that *C*. *arcuatus* and *C*. cf. *euspinulosum* are more abundant than *P*. *oulastreae* in Jeju waters. *C*. *arcuatus* and *C*. cf. *euspinulosum* are specialists in Jeju waters, inhabiting only the corals *M*. *millepora* and *A*. *japonica*, respectively. *C*. *arcuatus* is a generalist near Taiwan, where it covers a wider range of coral hosts. For example, in Taiwan waters [10, 11], *C*. *arcuatus* was recorded from corals such as *Montipora* spp. and *Porites* spp. This suggests that the host specificity of coral-associated barnacles varies with the availability of coral hosts and latitude. *C*. *arcuatus* appears to be a specialist in Jeju waters due to less choice in coral hosts, but it can be a generalist in subtropical and tropical waters with higher coral diversity due to more choice in coral hosts.

*C*. *euspinulosum* was named by Broch [33], based on *Cresusia spinulosa* variety 1, described by Darwin [34]. Broch’s specimens were found on madreporian corals, collected from Amboina and Jolo in Indonesian waters and Singapore. Since Broch, many researchers have reported *C*. *euspinulosum* from different locations in the Indo-Pacific. In the present study, DNA barcode sequences of *C*. cf. *euspinulosum* did not match the sequences available from GenBank and from the author’s collections of *Cantellius* specimens collected from Taiwan, Malaysia, Japan and Papua New Guinea. In this study’s molecular phylogenetic analysis, the DNA barcode sequences of *C*. cf. *euspinulosum* were clustered in a different clade from that of the *C*. *euspinulosum* reported by Tsang *et al*. [13] and Chan *et al*. [10, 11], which mainly inhabits *Porites* corals in Taiwan. The rostral tooth in the scutum of *C*. *euspinulosum* was not obvious and the scutum had no adductor plate in the study by Chan *et al*. [10, 11]. By contrast, the rostral tooth was obvious and an adductor plate was present in *C*. cf. *euspinulosum*. Results from the present study suggest that *C*. cf. *euspinulosum* is a cryptic species complex. Without molecular information on *C*. *euspinulosum* from the type locality in Indonesian waters, we cannot ascertain whether the specimen collected in Jeju waters is a new species. We therefore name the specimens collected as *C*. cf. *euspinulosum*. Further studies should collect *C*. *euspinulosum* from the type locality and compare the genetic differences among populations from different geographical locations to ascertain the taxonomic status of *C*. cf. *euspinulosum*.

The morphology of *C*. cf. *euspinulosum* is very similar to the illustration of *C*. *euspinulosum* from Palao provided by Hiro, 1936. According to the coral host ranges described by Hiro, 1936, *C*. *euspinulosum* in Palao was found only on the coral *Alveopora verriliana*. This suggests that *C*. cf. *euspinulosum* is a specialist, found only on *Alveopora* corals.

*P*. *oulastreae* has a relatively wider host usage that includes *Psammocora* and *Oulastrea* corals. Utinomi [35] also recorded a population of *P*. *oulastreae* that inhabits the coral *O*. *crispata* in Wakayama, Japan. In addition to the zooxanthellate coral hosts *Psammocora* and *Oulastrea*, Kim [19] described a population of *P*. *oulastreae* from Busan and Jeju waters that inhabited nonzooxanthellate corals. *P*. *oulastreae* appears to be a cold water species and is present at the extreme distribution limit of corals. *P*. *oulastreae* has been recorded from northern Honshu to Kyushu in Japan and at Jeju Island in the present study. According to the coral-associated barnacle survey by Asami and Yamaguchi [17], *P*. *oulastreae* is absent from Okinawa waters. Foster [14] recorded *P*. *oulastreae* from Hong Kong that inhabited *O*. *crispata* and the nonzooxanthellate corals *Tubastrea* and *Dendrophyllia*. However, Foster [14] did not provide a detailed description of this species. The taxonomic status of *Pyrgomina* from Hong Kong will require further morphological and molecular analysis. Molecular analysis in the present study revealed that *P*. *oulastreae* is located in a major clade with *Adna* and *Megatrema*. This is additional evidence that Megatrematinae forms a monophyletic unit.

Coral-associated barnacles in marginal communities have much lower diversity than their subtropical and tropical counterparts. When host availability is limited and lacks much choice in coral hosts, the host usage of marginal coral-associated barnacles is highly specific compared with that of barnacles in subtropical and tropical reef systems that have multiple choices for coral hosts from higher coral diversity. Global climatic changes engender modifications or declines in the coral host assemblages in marginal coral communities. This can have a substantial effect on the diversity of coral-associated barnacles, considering that they have a narrow host range in the marginal coral region.

## Supporting information

S1 File Taxonomic description of coral barnacles in Jeju waters. (PDF)

S2 File Trees resulting from single gene phylogenetic analyses. (PDF)

S1 Table Information on collection sites and GenBank no. for the sequences used for the phylogenetic analysis. (PDF)

## References

[1] Stella JS, Pratchett MS, Hutchings PA, Jones GP. Coral-associated invertebrates: diversity, ecological importance and vulnerability to disturbance. Oceanography and Marine Biology: An Annual Review. 2011;49:43–104.

[2] Blackall LL, Wilson B, Oppen MJH. Coral-the world’s most diverse symbiotic ecosystem. Molecular Ecology. 2015; 24(21):5330–5347. doi: 10.1111/mec.13400 26414414 10.1111/mec.13400

[3] Veron JEN. Corals of the World. Australian Inst Mar Sci 2000;1–3:1,382.

[4] De Palmas S, Denis V, Ribas-Deulofeu L, Loubeyres M, Woo S, Hwang SJ, et al *Sympobiodinium* spp. associated with high latitude scleractinian corals from Jeju Island, South Korea. Coral Reefs.2015;34(3):919–925.

[5] Denis V, Mezaki T, Tanaka K, Kuo CY, De Palmas S, Keshavmurthy S, et al Coverage, diversity, and functionality of a high-Latitude coral community (Tatsukushi, Shikoku Island, Japan). PLoS ONE. 2013;8(1): e54330 doi: 10.1371/journal.pone.0054330 23342135 10.1371/journal.pone.0054330 PMC3544760

[6] Sugihara K, Yamano H, Choi KS, Hyeong K. Zooxanthellate Scleractinian Corals of Jeju Island, Republic of Korea. Springer 2014:111–130.

[7] Ross A, Newman WA. Revision of the coral inhabiting barnacles (Cirripedia: Balanidae). Transactions of the San Diego Society of Natural History. 1973;17(12):137–174.

[8] Achituv Y, Mizrahi K. Recycling of ammonium within a hydrocoral (*Millepora dichotoma*)–zooxanthellae-cirripede (*Savignium milleporum*) symbiotic association. Bulletin of Marine Science. 1996;58(3):856–860.

[9] Achituv Y, Brickner I, Erez J. Stable carbon isotope ratios in Red Sea barnacles (Cirripedia) as an indicator of their food source. Marine Biology. 1997;130:243–247.

[10] Chan BKK, Chen YY, Achituv Y. Crustacean Fauna of Taiwan: Barnacles Volume II: Cirripedia: Thoracica: Pyrgomatidae. Biodiversity Research Center, Academia Sinica Press 2013a;367.

[11] Chan BKK, Chen YY, Lin HC. Biodiversity and host specificity of coral barnacles of *Galkinia* (Cirripedia: Pyrgomatidae) in Taiwan, with descriptions of six new species. Journal of Crustacean Biology. 2013b;33(3):392–431.

[12] Tsang LM, Chan BKK, Shih FL, Chu KH, Chen CLA. Host associated speciation in the coral barnacle *Wanella milleporae* (Cirripedia: Pyrgomatidae) inhabiting the *Millepora* coral. Molecular Ecology. 2009;18:1463–1475. doi: 10.1111/j.1365-294X.2009.04090.x 19368648 10.1111/j.1365-294X.2009.04090.x

[13] Tsang LM, Chu KH, Nozawa Y, Chan BKK. Morphological and host specificity evolution in coral symbiont barnacles (Balanomorpha: Pyrgomatidae) inferred from a multi-locus phylogeny. Molecular Phylogenetics and Evolution. 2014;77:11–22. doi: 10.1016/j.ympev.2014.03.002 24636895 10.1016/j.ympev.2014.03.002

[14] Foster BA. Shallow water barnacles from Hong Kong. Proceedings of the First International Marine Biological Workshop: The Marine Flora and Fauna of Hong Kong and southern China, Hong Kong, 1980. B. Morton (Ed). 1982:207–232.

[15] Chan BKK, Kolbasov G, Hirose M, Mezaki T, Suwae R. Biodiversity and biogeography of the coral boring barnacles of the genus *Berndtia* (Cirripedia: Acrothoracica) in the West Pacific, with description of three new species. Journal of Natural History. 2014;48(25–26):1503–1541.

[16] Rosell NC. Crustacea: Cirripedia. Résultats des campagnes MUSORSTOM I PHILIPPINES (18–28 MARS 1976) Ēditions de 1΄Office de la Recherche Scientifique et Technique Outre-Mer avec le concours du Muséum National d΄Histoire Naturelle. Série A, Zoologie. 1981;91:278–307.

[17] Asami K, Yamaguchi T. Distribution of living and fossil coral barnacles (Cirripedia; Pyrgomatidae) in Japan. Sessile Organisms. 1997;14(1):9–16.

[18] Choi E, Song JI. Four new records of two genera *Balanophyllia* and *Cladopsammia* (Anthozoa: Hexacorallia: Scleractinia: Dendrophylliidae) from Korea. Animal Systematics, Evolution and Diversity. 2014;30(3):183–190.

[19] Kim IH. Barnacles. National Institute of Biological Resources, Ministry of Environment, South Korea. vertebrate Fauna of Korea. 2011;21(6):105–108.

[20] Chan BKK, Garm A, Høeg JT. Setal morphology and cirral setation of thoracican barnacle cirri: adaptations and implications for thoracican evolution. Journal of Zoology. 2008;275(3):294–306. doi: 10.1111/j.1469-7998.2008.00441.x

[21] Chen HN, Tsang LM, Chong VC, Chan BKK. Worldwide genetic differentiation in the common fouling barnacle, *Amphibalanus amphitrite*. The Journal of Bioadhesion and Biofilm Research. 2014;30(9):1067–1078. 10.1080/08927014.2014.967232 25343722

[22] Achituv Y, Tsang LM, Chan BKK. A new species of *Cantellius* and redescription of C *sextus* (Hiro, 1938) (Cirripedia, Balanomorpha Pyrgomatidae) from the elephant skin coral, *Pachyseris speciosa* (Dana, 1846) (Scleractinia, Agariciidae) from Taiwan. Zootaxa. 2009;2022:15–58.

[23] Chan BKK, Chen YY, Lin HC. Biodiversity and host specificity of coral barnacles of Galkinia (Cirripedia: Pyrgomatidae) in Taiwan, with descriptions of six new species. Journal of Crustacean Biology. 2013;33:392–431. doi: 10.1163/1937240X-00002134

[24] Chan BKK, Liu JCW. *Galkinius* Perreault, 2014 or *Darwiniella* (Anderson, 1992)? A new coral-associated barnacle sharing characteristics of these two genera in Pacific waters (Crustacea, Cirripedia, Thoracica, Pyrgomatidae). ZooKeys. 2017;719:1–22. doi: 10.3897/zookeys.719.12471 29290719 10.3897/zookeys.719.12471 PMC5740467

[25] Kumar S, Stecher G, Tamura K. MEGA7: Molecular Evolutionary Genetics Analysis Version 7.0 for Bigger Datasets. Molecular Biology and Evolution. 2016;33(7):1870–1874. doi: 10.1093/molbev/msw054 27004904 10.1093/molbev/msw054 PMC8210823

[26] Katoh K, Misawa K, Kuma K, Miyata T. MAFFT: a novel method for rapid multiple sequence alignment based on fast Fourier transform. Nucleic Acids Research. 2002;30(14):3059–3066. doi: 10.1093/nar/gkf436 12136088 10.1093/nar/gkf436 PMC135756

[27] Stamatakis A. RAxML version 8: a tool for phylogenetic analysis and post-analysis of large phylogenies. Bioinformatics. 2014;30(9):1312–1313. doi: 10.1093/bioinformatics/btu033 24451623 10.1093/bioinformatics/btu033 PMC3998144

[28] Asami K, Yamaguchi T. Distribution of living and fossil coral barnacles (Cirripedia; Pyrgomatidae) in Japan. Sessile Organisms. 1997;14:9–16.

[29] Ogawa K, Matsuzaki K. Revision of the coral-inhabiting barnacles in Japan—preliminary note. Nanki-Seibutu, Nanki Biological Society.1990; 32:73–79.

[30] Chan BKK, Chen YY, Achituv Y. Crustacean Fauna of Taiwan: Barnacles Volume II: Cirripedia: Thoracica: Pyrgomatidae. Biodiversity Research Center, Academia Sinica Press 2013a;1–364.

[31] Ministry of the Environment. Coral Reefs of Japan. Ministry of Environment, Japan 2004;1–359.

[32] Dai CF, Horng S. Scleractinia of Taiwan I. The Complex Group. National Taiwan University 2009;172.

[33] Broch H. Papers from Dr. Th. Mortensen’s Pacific Expedition 1914–16. LVI. Indomalayan Cirripedia. Videnskabelige meddelelser fra den Naturhistoriske forening Kjobenhavn.1931;91:1–146.

[34] Darwin C. A monograph on the sub-class Cirripedia with figures of all the species The Balanidae, the Verrucidae, etc. Royal Society, London 1854;684.

[35] Utinomi H. Studies on the Cirripedian fauna of Japan. VIII. Thoracic cirripeds from western Kyushu. Publications of the Seto Marine Biological Laboratory. 1962;10:211–239.

