# Supplementary material for: Living with marginal coral communities: Diversity and host-specificity in coral-associated barnacles in the northern coral distribution limit of the East China Sea
Source: PLoS One. 2018 May 1;13(5):e0196309. doi: 10.1371/journal.pone.0196309 (PMC5929504; doi:10.1371/journal.pone.0196309)
Supplement: S1 File — (PDF) [file pone.0196309.s001.pdf]

Taxonomic description of coral barnacles in Jeju waters

Family Pyrgomatidae Gray, 1825

Subfamily Pyrgomatinae Gray, 1825

Cantellius arcuatus Hiro, 1938

S1A and S1B and S2A-S2D and S3-S6 Figs

Creusia spinulosa forma *arcuata* Hiro, 1938: 395, 403, fig 3a-c, tab. II. [1] —Kolosváry, 1947a: 426, 428. [2] —Kolosváry, 1947b: 364, 367. [3] —Utinomi, 1949a: 69. [4]

Cantellius arcuatum. —Ross and Newman, 1973: 150, fig. 7d-e. [5] —Newman and Ross, 1976: 57. [6] —Foster, 1980: tab. 5. [7] —Ogawa and Matsuzaki, 1990: tab. 1. [8]

Cantellius arcuatus.—Galkin, 1986: 1290. [9] —Ogawa and Matsuzaki, 1992: app. tab. [10] —Asami and Yamaguchi, 1997: 14, figs 1-2. [11] —Ogawa et al., 1998: 12, fig 11. [12] —Chan et al. 2013a: 12, figs 23-31. [13]

Materials examined. CEL-JJ-01, 211 specimens (BD 2.02 – 5.47 mm), 33°13.917' N 126°35.800' E, Seop Seom (NW), Jeju Island, Korea, 6 August 2016, coll. B.K.K. Chan, on host coral *Montipora millepora*. CEL-JJ-02, 142 specimens (BD 1.29 – 7.18 mm), same data as CEL-JJ-01. CEL-JJ-13, 130 specimens (BD 2.53 – 8.05 mm), same data as CEL-JJ-01. CEL-JJ-14, 36 specimens (BD 3.01 – 5.98 mm), same data as CEL-JJ-01. CEL-JJ-15, 96 specimens (BD 2.16 – 7.59 mm), same data as CEL-JJ-01. CEL-JJ-16, 59 specimens (BD 2.43 – 8.00 mm), Seop Seom (NE), Jeju Island, Korea, 7 August 2016, coll. B.K.K. Chan, on host coral *Montipora millepora*. CEL-JJ-17, 29 specimens (BD 3.46 – 5.65 mm), 33°13.839' N, 126°36.155' E, Seop Seom (SE), Jeju Island, Korea, 7 August 2016, coll. B.K.K. Chan, on host coral *Montipora millepora*. CEL-JJ-18, 80 specimens (BD 2.46 – 8.53 mm), same data as CEL-JJ-16. CEL-JJ-19, 44 specimens (BD 2.34 – 4.34 mm), same data as CEL-JJ-16. CEL-JJ-20, 73 specimens (BD 1.93 – 5.87 mm), same data as CEL-JJ-16. CEL-JJ-23, 88 specimens (BD 1.76 – 7.02 mm), same data as CEL-JJ-17. CEL-JJ-25, 120 specimens (BD 2.436 – 9.70 mm), same data as CEL-JJ-17. CEL-JJ-28, 11 specimens (BD 2.11 – 6.16 mm), 33°07.214' N, 126°15.821' E, Marado (N), Jeju Island, Korea, 9 August 2016, coll. B.K.K. Chan, on host coral *Montipora millepora*. CEL-JJ-33, 70 specimens (BD 2.80 – 3.80 mm),

same data as CEL-JJ-16. CEL-JJ-34, 43 specimens (BD 2.82 – 6.97 mm), same data as CEL-JJ-17. CEL-JJ-35, 44 specimens (BD 3.62 – 8.79 mm), 33°13.115' N, 126°30.850' E, Beom Seom, Jeju Island, Korea, 10 August 2016, coll. B.K.K. Chan, on host coral *Montipora millepora*. CEL-JJ-36, 14 specimens (BD 4.99 – 7.49 mm), Beom Seom, Jeju Island, Korea, 10 August 2016, coll. B.K.K. Chan, on host coral *Montipora millepora*. CEL-JJ-38, 58 specimens (BD 3.89 – 6.73 mm), same data as CEL-JJ-36. CEL-JJ-41, 142 specimens (BD 1.52 – 5.96 mm), same data as CEL-JJ-36. CEL-JJ-43, 28 specimens (BD 2.72 – 6.65 mm), 33°13.345' N 126°30.753' E, Beom Seom (NW), Jeju Island, Korea, 11 August 2016, coll. B.K.K. Chan, on host coral *Montipora millepora*.

Diagnosis. Shell composed of 4 separated plates. External surfaces of apex in scutum and tergum with honeycomb-like structure. Occludent margin of scutum straight, without a rostral tooth. Adductor plate absent in scutum. Tergum triangular, spur distinct, long and narrow. Medial furrow curved. Posterior side of segments on Cirri III with large spines.

Description. Shell ovate, white, 4-plated (rostrum, carina, and paired laterals). External surface covered by coral tissue (S1A and S1B and S2A Figs). Base of shell with approximately 33 longitudinal septa radiating from rim of sheath to external surface of shell (8 in rostrum and 10 in carina, 8 in laterals, (S2B and S3A Figs), septa margin serrated (S2B and S3A Figs). Orifice ovate, about 1/4 basal rostro-carinal diameter (S2A Fig). Scutum and tergum separated (S2C and S2D and S3B–S3E Figs), white. Scutum triangular, width equal to height, occludent margin straight, without a rostral tooth, 12-14 teeth along occludent margin, tooth size increasing gradually from apex to base margin (S2D and S3B Figs). Scutum apex with honeycomb-like pattern (S2C Fig). External scutum surface with multiple horizontal striations. Internal view with an oval-shaped adductor muscle scar (S3C Fig). Adductor plate absent. Basal margin of scutum with a notch near tergal margin (S2C and S2D and S3B and S3C Figs). Tergum triangular. Spur distinct, long and narrow (S2C and S2D and S3D and S3E Figs). Tergal apex curved toward scutal margin (S2C and S2D and S3D and S3E Figs). External surface with a shallow medial furrow, curving from basal margin towards carinal margin, width of furrow increasing gradually from apex to base (S2C and S3D Figs). External surface with horizontal striations, striations with row of small pores (S2C and S3D Figs).

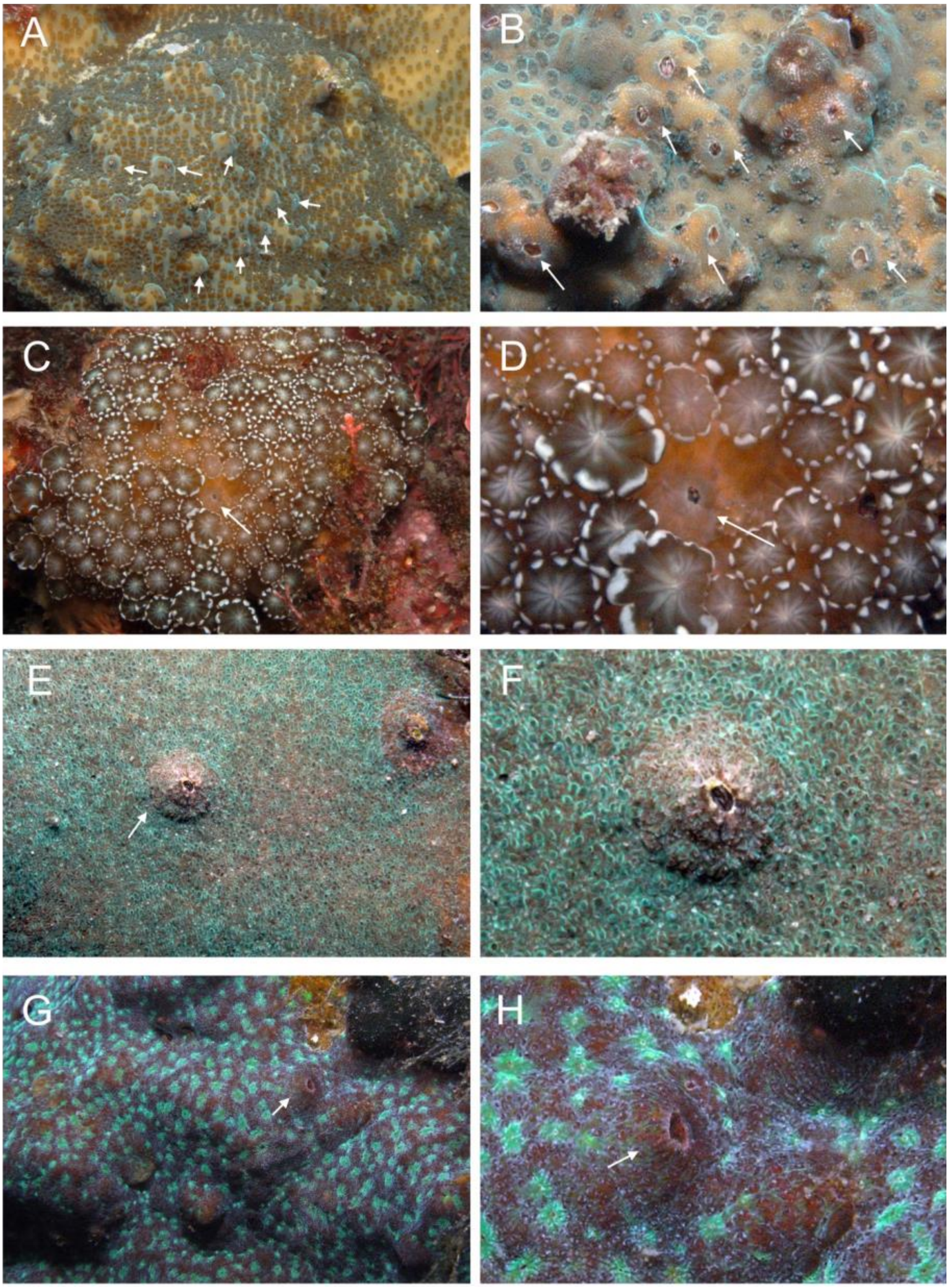

S1 Fig. In-situ underwater photos of coral associated barnacles in Jeju waters. A, The coral *Montipora millepora*, colonized by the barnacle *Cantellius arcuatus* (indicated by white arrows). B, Close up view of *Cantellius arcuatus*, indicated by white arrows. C, The coral *Alveopora japonica* inhabited by a single individual of *Cantellius* cf. *euspinulosum*, indicated by white arrow. D, Close up view of *Cantellius* cf. *euspinulosum*. E, *Psammocora* coral inhabited by the barnacle *Pyrgomina*

oulastreae. F, Close up view of *Pyrgomina oulastreae* on coral *Psammocora*. G, The coral *Oulastrea crispata* inhabited by the barnacle *Pyrgomina oulastreae*. H, Close up view of *Pyrgomina oulastreae* on coral *Oulastrea crispata*.

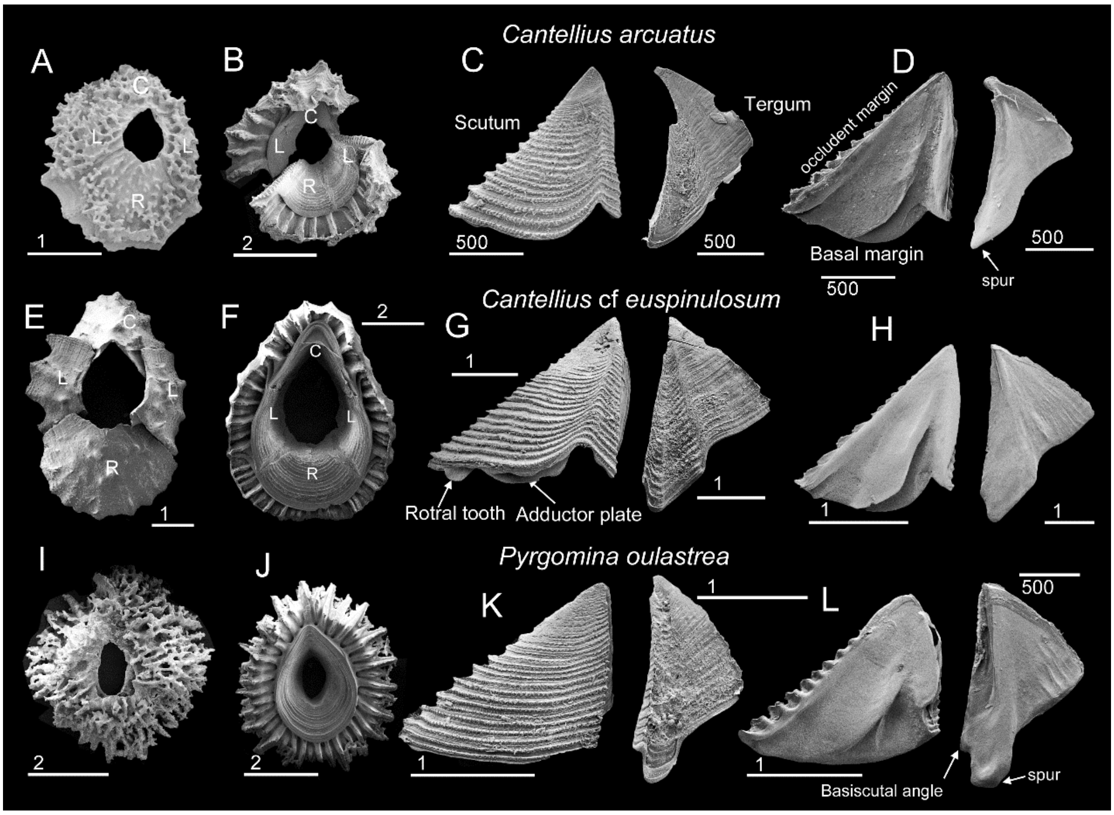

S2 Fig. Scanning electron microscopy on the hard parts of coral associated barnacles in Jeju Island. A, External and B, Internal view of the shell of *Cantellius arcuatus*, showing the 4-plated shell (C – carina, L – laterals, R – rostrum). C, External view of scutum and tergum of *C. arcuatus*. D, Internal view of *C. arcuatus*. E, External and F, Internal view of scutum and tergum of *Cantellius* cf. *euspinulosum*. G, External view of scutum and tergum of *C.* cf. *euspinulosum*, showing the rostral tooth and adductor plate on scutum. H, Internal view of scutum and tergum of *C.* cf. *euspinulosum*. I, External and J, Internal view of *Pyrgomina oulastreae*, showing a single fused shell. K, External view of scutum and tergum of *Pyrgomina oulastreae*. L, Internal view of scutum and tergum of *Pyrgomina oulastreae*.

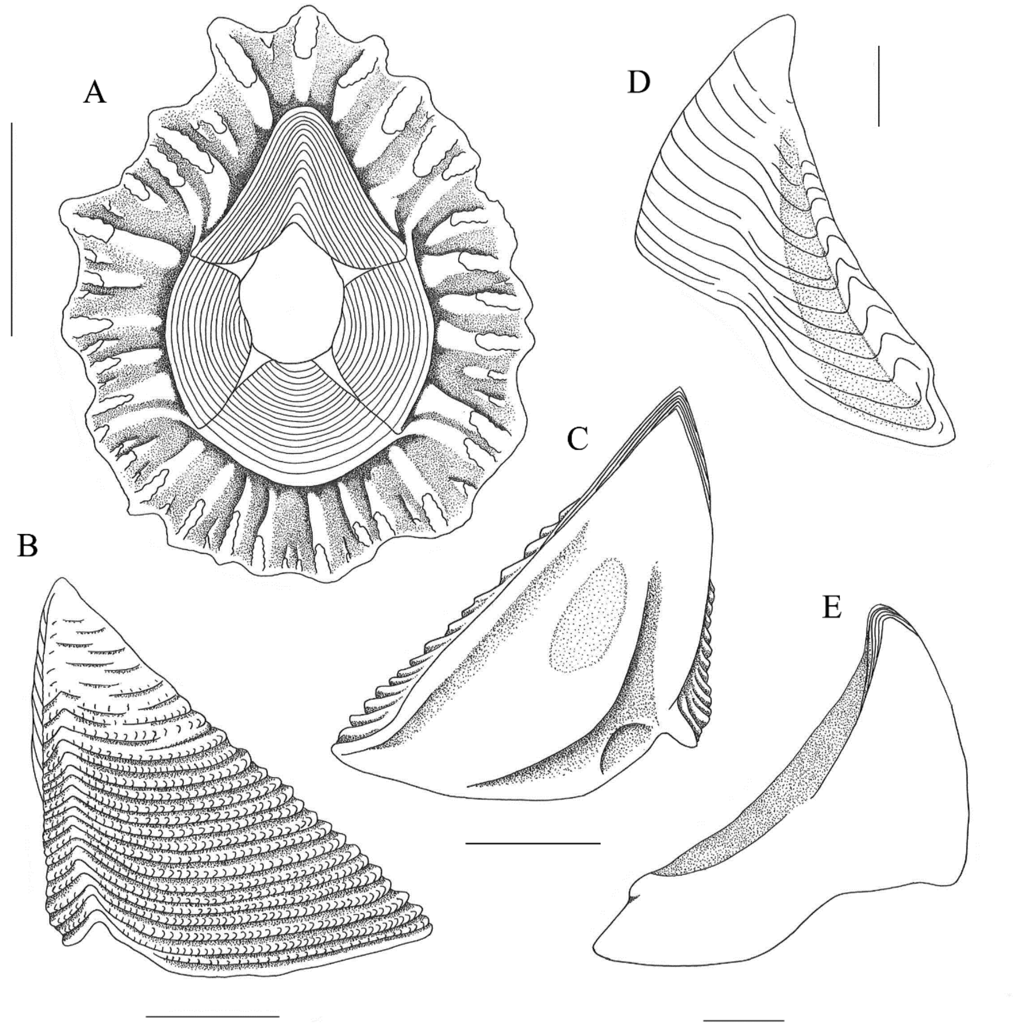

S3 Fig. *Cantellius arcuatus*. Line drawing showing. A, Internal view of shell. B, External view of scutum. C, Internal view of scutum. D, External view of tergum. E, Internal view of tergum. Scale bars: A=1.27 mm, B, C=0.8mm, D, E=0.5 mm.

Maxilla ovate (S4A and S5A and S5B Figs), with serrulate-type setae distally (S5B Fig). Maxillule cutting edge straight without notch, bearing row of 8-9 large setae (S4C and S5C and S5D Figs). Region close to cutting edge with dense fine simple-type setae, anterior and posterior margins with long simple-type setae (S4C and S5C and S5D Figs). Mandible with 5 teeth, excluding inferior angle (3 specimens, S4F and S5E and S5F Figs). Second to fourth teeth bidentate (S4F and S5E and S5F Figs), the first 3 teeth occupied 3/4 length of cutting edge. Lower margin short, inferior angle blunt with simple-type setae (S4F and S5E and S5F Figs). Mandibular palp subtriangular (S4B and S5G Figs), bearing serrulate setae distally (S5G Fig) and on interior margin (S5G Fig). Labrum bilobed, lobes separated by a V-shaped notch, 2-4 sharp teeth on each side of notch (S4E and S5H Figs).

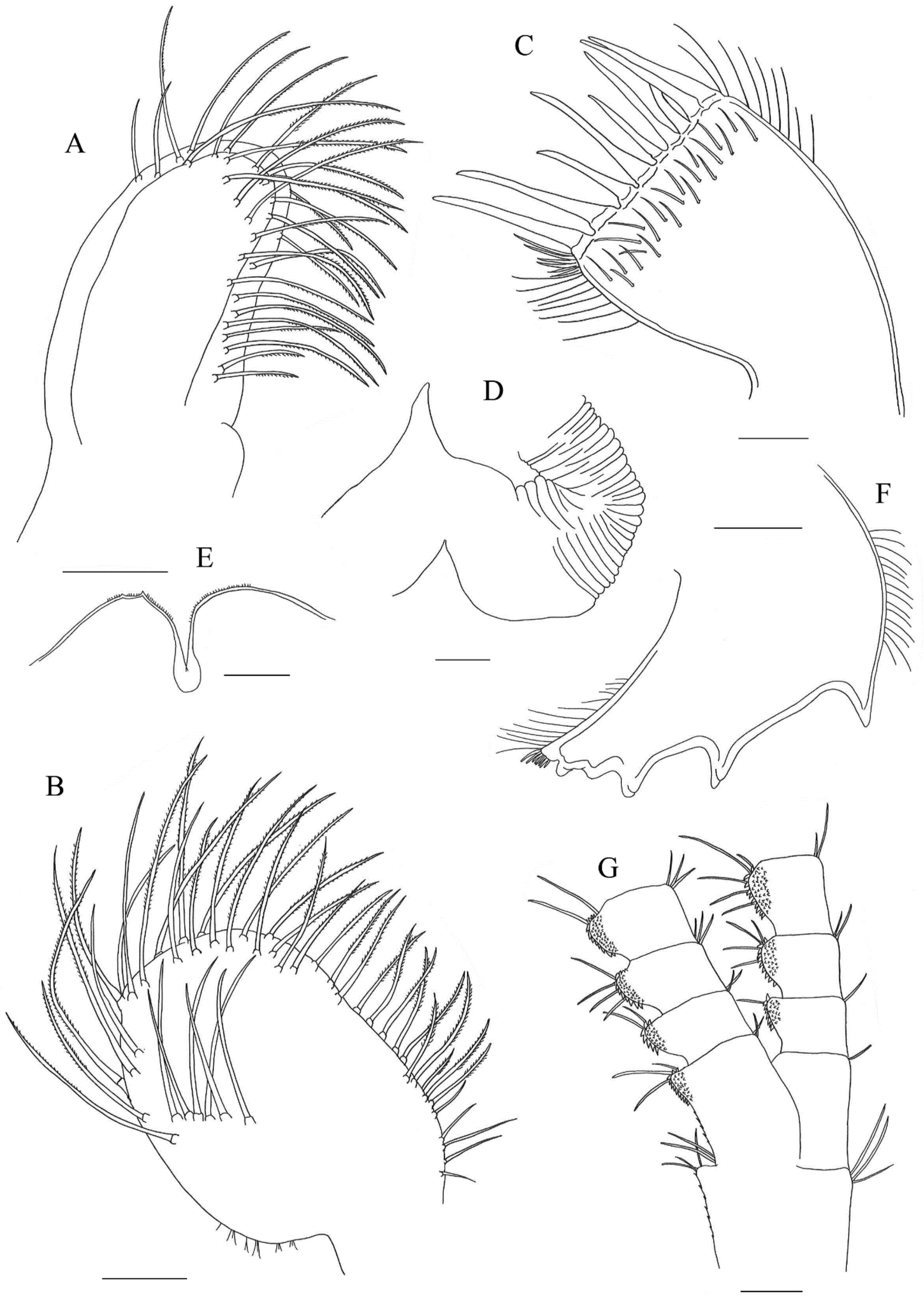

S4 Fig. ***Cantellius arcuatus*****.** Line drawing showing. A, Maxilla, B, Mandibulatory palp. C, Maxillule. D, Basi-dorsal point of penis. E, Labrum. F, Mandible. G, Intermediate segment of cirrus IV, showing the spines. Scale bars: A–G=0.1 mm.

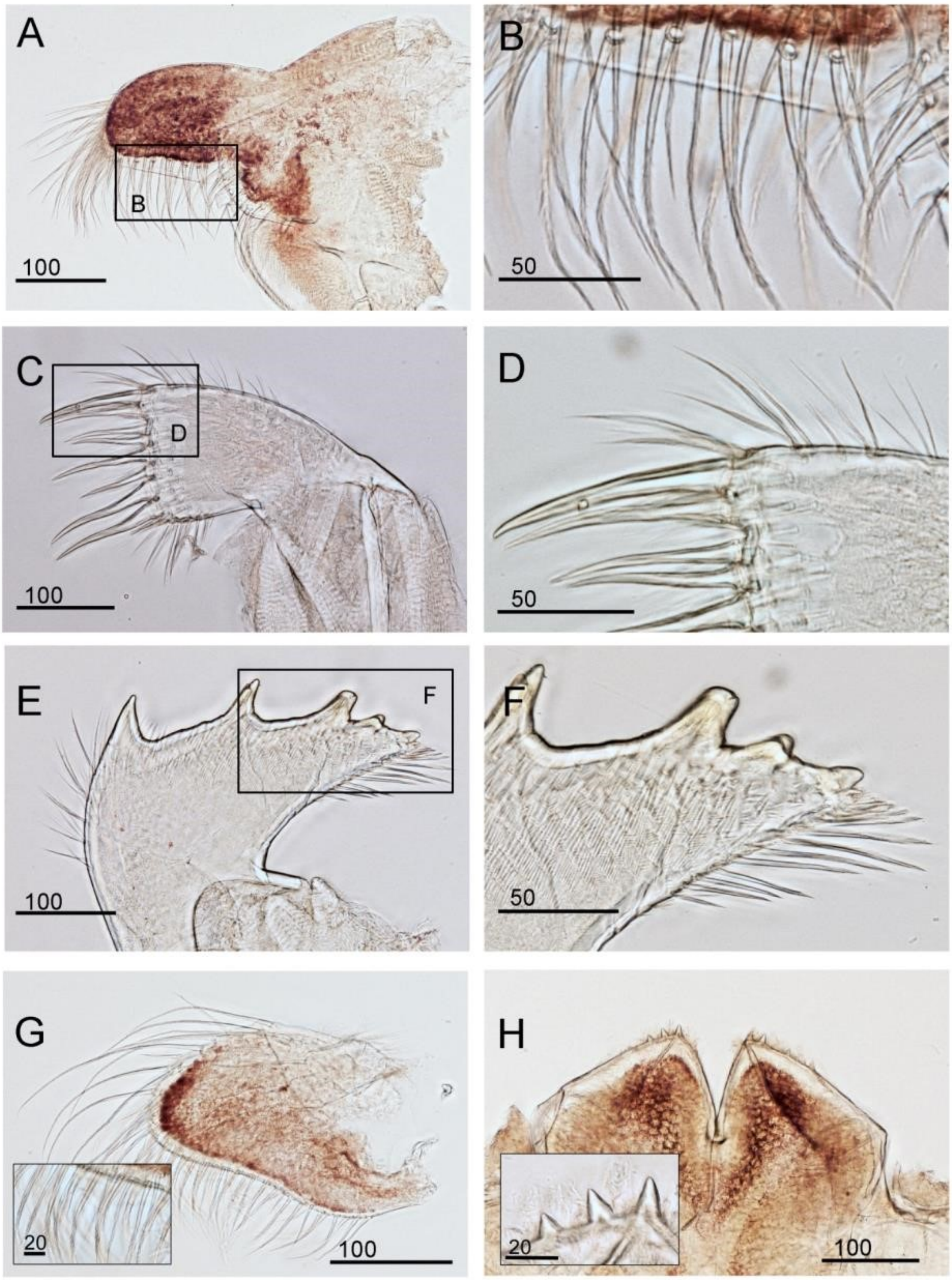

S5 Fig. *Cantellius arcuatus*. Light microscopy showing. A, Maxilla. B, Serrulate setae on margin of maxilla. C, Maxillule. D, Cutting margin and external margin of maxillule showing simple type setae. E, Mandible. F, Second, third, fourth and fifth teeth. G, Mandibulatory palp. H, Labrum. Scale bars in μm.

Cirrus I with unequal rami, anterior ramus long, slender, 10-segmented, posterior

ramus 5-segmented (S6A Fig), bearing serrulate setae (S6A Fig). Cirrus II (S6B Fig) anterior ramus with 7-segments, slightly longer than posterior ramus (5-segmented), bearing serrulate setae (S6B Fig). Cirrus III anterior ramus 8-segmented, posterior ramus 6-segmented (S6C Fig), bearing serrulate setae, with sharp teeth on base of each segment (S6D Fig). Cirri IV-VI very long, slender, rami equal (S6E-S6G Figs). Cirrus IV with anterior ramus 14-segmented, posterior ramus 15-segmented. Cirrus V (anterior 15-segmented, posterior 19-segmented), Cirrus VI (anterior 14-segmented, posterior 17-segmented). Each intermediate segment of ramus of Cirrus IV - VI with 1 pair of long serrulate and 1 pair simple setae (S6E-S6G Figs). Penis length equal to length of Cirrus VI, annulated, with scattered short simple-type setae (S6H Fig). Pedicel with a sharp basidorsal point (S6H Fig).

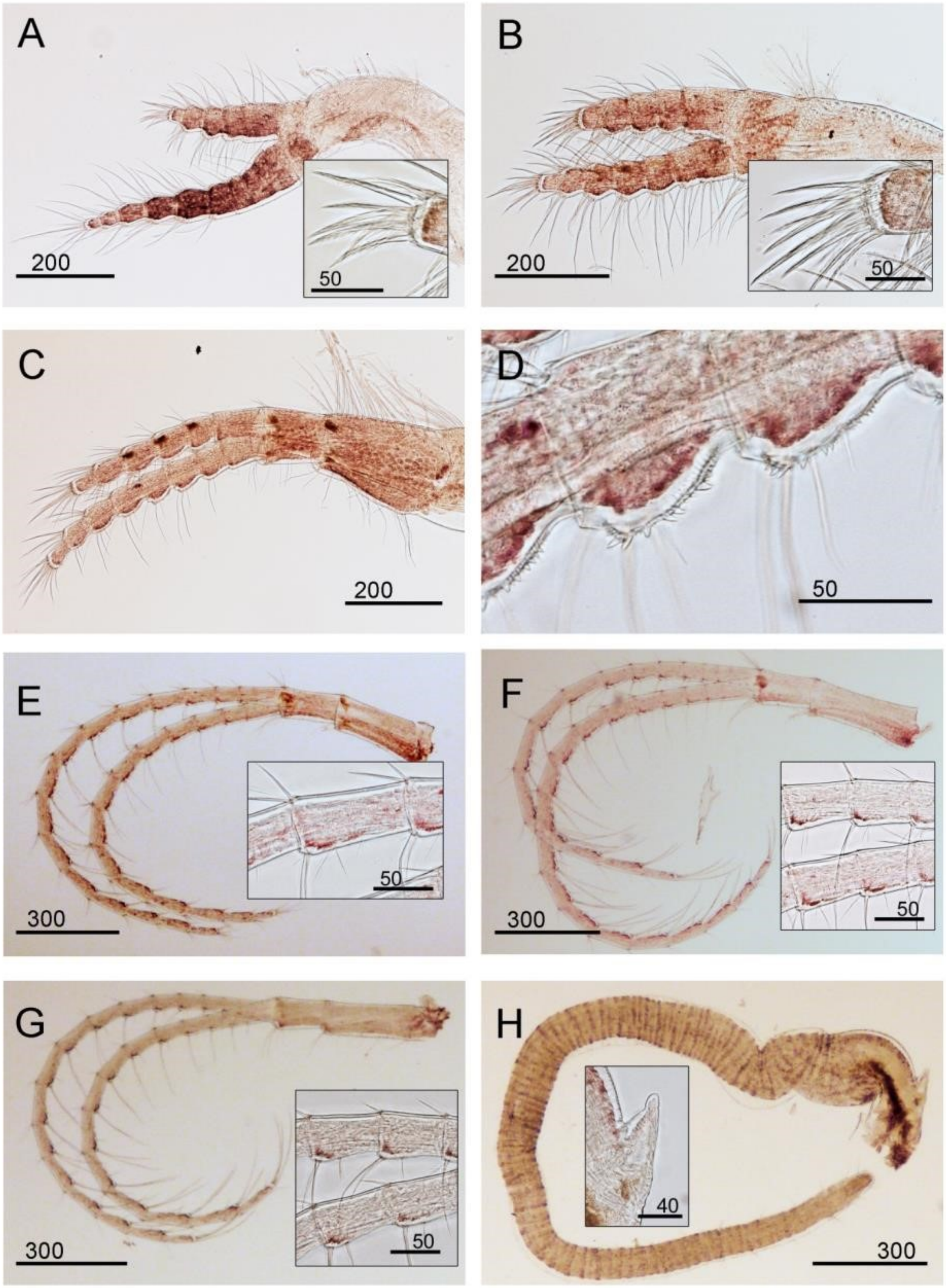

S6 Fig. ***Cantellius arcuatus*****.** Light microscopy on A, Cirrus I. B, Cirrus II. C, Cirrus III. D, Magnified view of posterior ramus, showing the hook-shaped spines. E, Cirrus IV. F, Cirrus V. G, Inserts in E–G showing the intermediate segments. H, Penis. Insert showing the basi-dorsal point of penis. Scale bars in μm.

Habitat. Jeju Island: Recorded on host coral *Montipora millepora* exclusively. Other regions: recorded on host coral includes *Acropora pruinosa* Brook, 1893, *Aropora* spp., *Coeloseris mayeri* Vaughan, 1918, *Montipora foliosa* Pallas, 1766, *M. prolifera* Brüggemann, 1879, *M.* spp., *Pavona crassa* Dana, 1840, *P. lata* Dana, 1846, *Platygyra* sp., *Porites capricornis* Rehberg, 1891, *Porites* spp., *Psammocora contigua* Esper, 1797.

Distribution. Jeju Island, Korea, Palau Island, Mauritius, Hong Kong, Japan, Taiwan, Papua New Guinea and Malaysia.

Remarks. *Cantellius arcuatus* Hiro, 1938 is the first report in the present study from Korean waters. The genus *Cantellius* Ross and Newman [5] have been recorded 26 species in the world. Of these, only two species have been reported in Korea from Jeju Island. This species can be distinguished from *C. euspinulosum* by scutum. *C. arcuatus* has no rostral tooth in basal margin while *C. euspinulosum* has rostral tooth.

Cantellius cf. *euspinulosum* Broch, 1931

S1C and S1D and S2E-S2H and S7-S10 Figs

Pyrgoma (*Creusia*) *spinulosa* var. 1.—Darwin, 1854: 377-378, pl. 13, figs 6a-d. [14]

Creusia spinulosa var. VI. —Gruvel, 1905: 299, fig 323. [15]

Creusia spinulosa forma *eu-spinulosa* Broch, 1931: 118. [16] —Nilsson-Cantell, 1938:
59, text fig 20. [17] —Hiro, 1938: 393, fig 1a-c. [1]

Creusia spinulosa var. *eu-spinulosa*.—Hiro, 1935: 48-50, fig 1. [18]

Cantellius euspinulosa.—Jones et al., 2000: 273. [19] —Achituv and Newman, 2002:
392-393. [20]

Cantellius euspinulosum.—Ross and Newman, 1973: 150, fig. 7g-i. [5] —Newman and
Ross, 1976: 57. [6] —Anderson, 1992: 289-292, figs 7-8. [21] —Paulay and Ross, 2003: 309. [22] —Ogawa and Tachikawa, 2009: 91, fig 10. [23] —Kim 2011:105, fig 57. [24] —Chan *et al*., 2013a: 22, figs 32-40. [13]

Cantellius euspinulosus. —Poltarukha and Dautova, 2007: 99, 101, fig 59. [25]

Materials examined. CEL-JJ-37, 7 specimens (BD 2.41 – 6.05 mm), Beom Seom, Jeju Island, Korea, 10 August 2016, coll. B.K.K. Chan, on host coral *Alveopora japonica*. CEL-JJ-39, 35 specimens (BD 1.70 – 4.78 mm), same data as CEL-JJ-37. CEL-JJ-45, 1 specimen (BD 7.87 mm), Yongsuri, Jeju Island, Korea, 9 August 2016,

coll. S. K. Choi, on host coral *Alveopora japonica*. CEL-JJ-46, 5 specimens (BD 1.28 – 6.65 mm), same data as CEL-JJ-45. CEL-JJ-47, 11 specimens (BD 3.03 – 7.16 mm), same data as CEL-JJ-45. CEL-JJ-48, 5 specimens (BD 4.49 – 7.21 mm), same data as CEL-JJ-45. CEL-JJ-49, 10 specimens (BD 2.04 – 6.95 mm), same data as CEL-JJ-45. CEL-JJ-50, 18 specimens (BD 3.23 – 9.35 mm), same data as CEL-JJ-45. CEL-JJ-51, 6 specimens (BD 4.15 – 5.82 mm), same data as CEL-JJ-45. CEL-JJ-52, 4 specimens (BD 3.23 – 5.66 mm), same data as CEL-JJ-45. CEL-JJ-53, 6 specimens (BD 4.54 – 6.11 mm), same data as CEL-JJ-45. CEL-JJ-54, 12 specimens (BD 3.16 – 7.92 mm), same data as CEL-JJ-45. CEL-JJ-55, 5 specimens (BD 2.88 – 6.72 mm), same data as CEL-JJ-45. CEL-JJ-56, 15 specimens (BD 3.68 – 6.08 mm), same data as CEL-JJ-45. CEL-JJ-57, 1 specimen (BD 7.05 mm), same data as CEL-JJ-45. CEL-JJ-58, 3 specimens (BD 2.36 – 5.35 mm), same data as CEL-JJ-45. CEL-JJ-59, 3 specimens (BD 3.35 – 4.54 mm), same data as CEL-JJ-45. CEL-JJ-60, 1 specimen (BD 4.63 mm), same data as CEL-JJ-45. CEL-JJ-61, 9 specimens (BD 3.82 – 6.33 mm), same data as CEL-JJ-45. CEL-JJ-62, 2 specimens (BD 4.86 – 5.42 mm), same data as CEL-JJ-45. CEL-JJ-63, 3 specimens (BD 4.47 – 6.22 mm), same data as CEL-JJ-45. CEL-JJ-64, 6 specimens (BD 4.56 – 7.15 mm), same data as CEL-JJ-45. CEL-JJ-65, 3 specimens (BD 3.81 – 5.31 mm), same data as CEL-JJ-45. CEL-JJ-66, 1 specimen (BD 5.93 mm), same data as CEL-JJ-45. CEL-JJ-67, 7 specimens (BD 3.75 – 6.05 mm), same data as CEL-JJ-45. CEL-JJ-68, 5 specimens (BD 4.09 – 6.56 mm), same data as CEL-JJ-45. CEL-JJ-69, 2 specimens (BD 4.73 – 6.54 mm), same data as CEL-JJ-45. CEL-JJ-70, 5 specimens (BD 3.32 – 9.09 mm), same data as CEL-JJ-45. CEL-JJ-71, 1 specimen (BD 5.61 mm), same data as CEL-JJ-45. CEL-JJ-72, 2 specimens (BD 3.87 – 5.07 mm), same data as CEL-JJ-45. CEL-JJ-74, 2 specimens (BD 2.67 – 3.59 mm), same data as CEL-JJ-45. CEL-JJ-75, 4 specimens (BD 4.24 – 7.99 mm), same data as CEL-JJ-45. CEL-JJ-76, 2 specimens (BD 4.70 – 6.20 mm), same data as CEL-JJ-45. CEL-JJ-08, 5 specimens (BD 5.71 – 7.36 mm), same data as CEL-JJ-45.

Diagnosis. Scutum triangular, width approximately equal to height, occludent margin straight, rostral tooth not apparent. Adductor plate and rostral tooth present. Tergum triangular, spur blunt and short. Medial furrow of tergum curved.

Description. Shell conical and ovate, 4-plated (rostrum, carina, and paired laterals). External surface covered by coral tissue (S1C and S1D and S2E and S2F and S7A Figs). Base of shell with approximately 29 longitudinal septa radiating from rim of sheath to external shell surface (8 in rostrum and carina, 6 and 7 in laterals, respectively; S7B Fig), septa margin serrated (S7B Fig). Orifice circular, about 2/5 length of rostro-carinal diameter. Scutum and tergum separated (S2G and S2H Figs), basically white, with purple color in apex region. Scutum triangular, width approximately equal to height, occludent margin straight, rostral tooth and adductor

plate present (S2G and S7C Figs). External surface with horizontal striations, striations with row of small pores (S2G and S7C Figs). Internal view with a deep depressor muscle crest (S2H and S7D Figs). Tergum triangular. Spur blunt, width of basal margin of tergum equal to height of tergum (S2H and S7E and S7F Figs). External surface with a shallow medial furrow, extending from basal margin towards apex, width of furrow increasing gradually from apex to base. External surface with horizontal striations (S7F Fig).

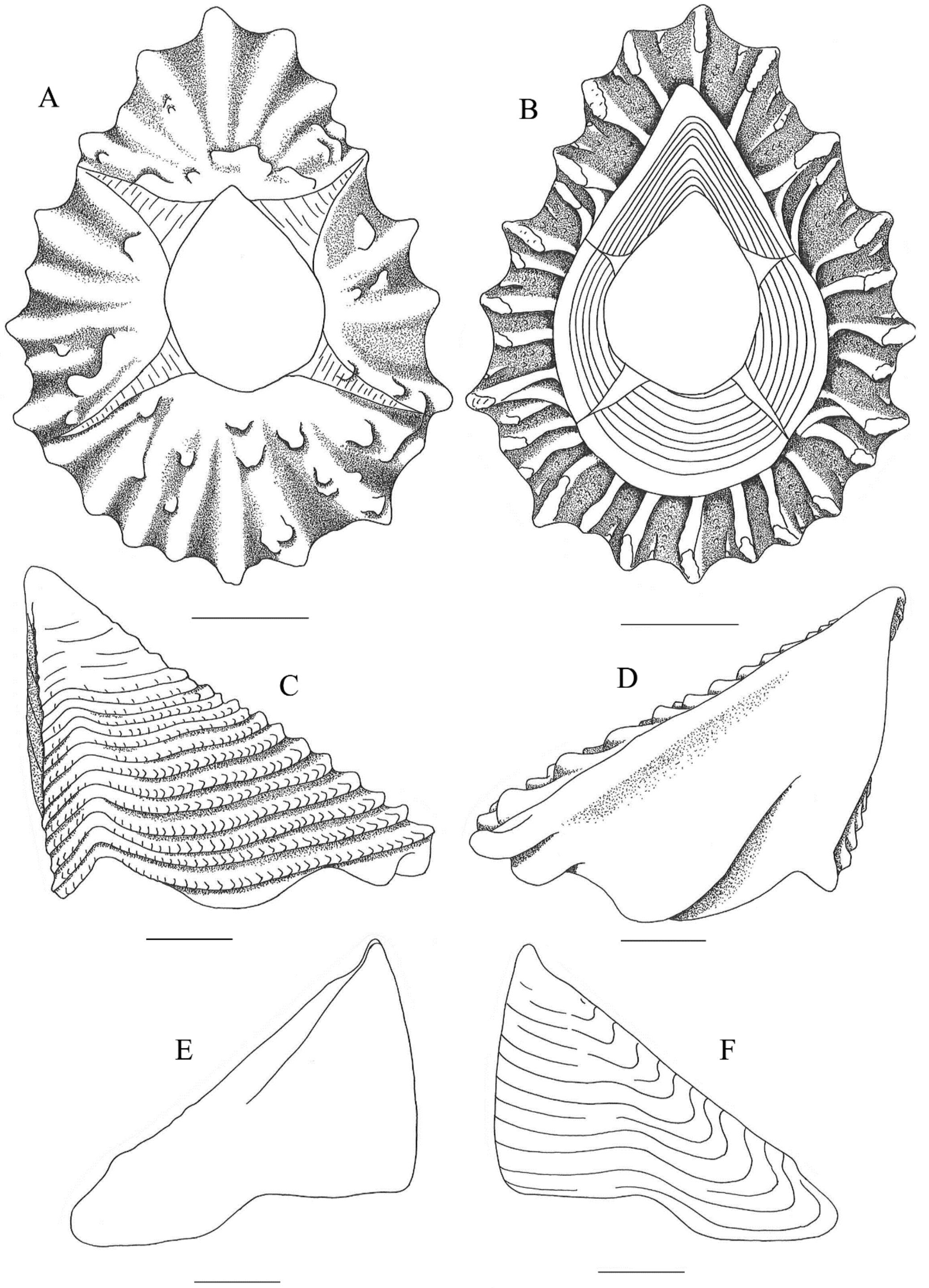

S7 Fig. *Cantellius* cf. *eusponulosum*. Line drawing showing. A, External view of shell. B, Internal view of shell. C, External view of scutum. D, Internal view of scutum. E, External view of tergum. F, Internal view of tergum. Scale bars: A, B=1 mm, C, D=0.45 mm, E, F=0.5 mm.

Maxilla ovate (S8A and S9A Figs), with serrulate setae on margin (S9B Fig). Maxillule cutting edge straight without notch, bearing row of 9 large setae (S8E and

S9C Figs). Region close to cutting edge with dense simple-type setae, anterior and posterior margins with simple-type setae (S9D Fig). Mandible with 5 teeth, excluding inferior angle (S8B and S9E and S9F Figs). Second to fourth teeth bidentate (S9E and S9F Figs), first 3 teeth occupied 4/5 length of cutting edge. Lateral surface, lower margin and cutting edge of mandible bearing simple-type setae. Lower margin short, inferior angle blunt with simple-type setae (S9F Fig). Mandibular palp elongated (S9G Fig), bearing serrulate setae distally and on interior margin (S9G Fig). Labrum bilobed, lobes separated by a V-shaped notch, 3 sharp teeth on each side of notch (S8D and S9H Figs).

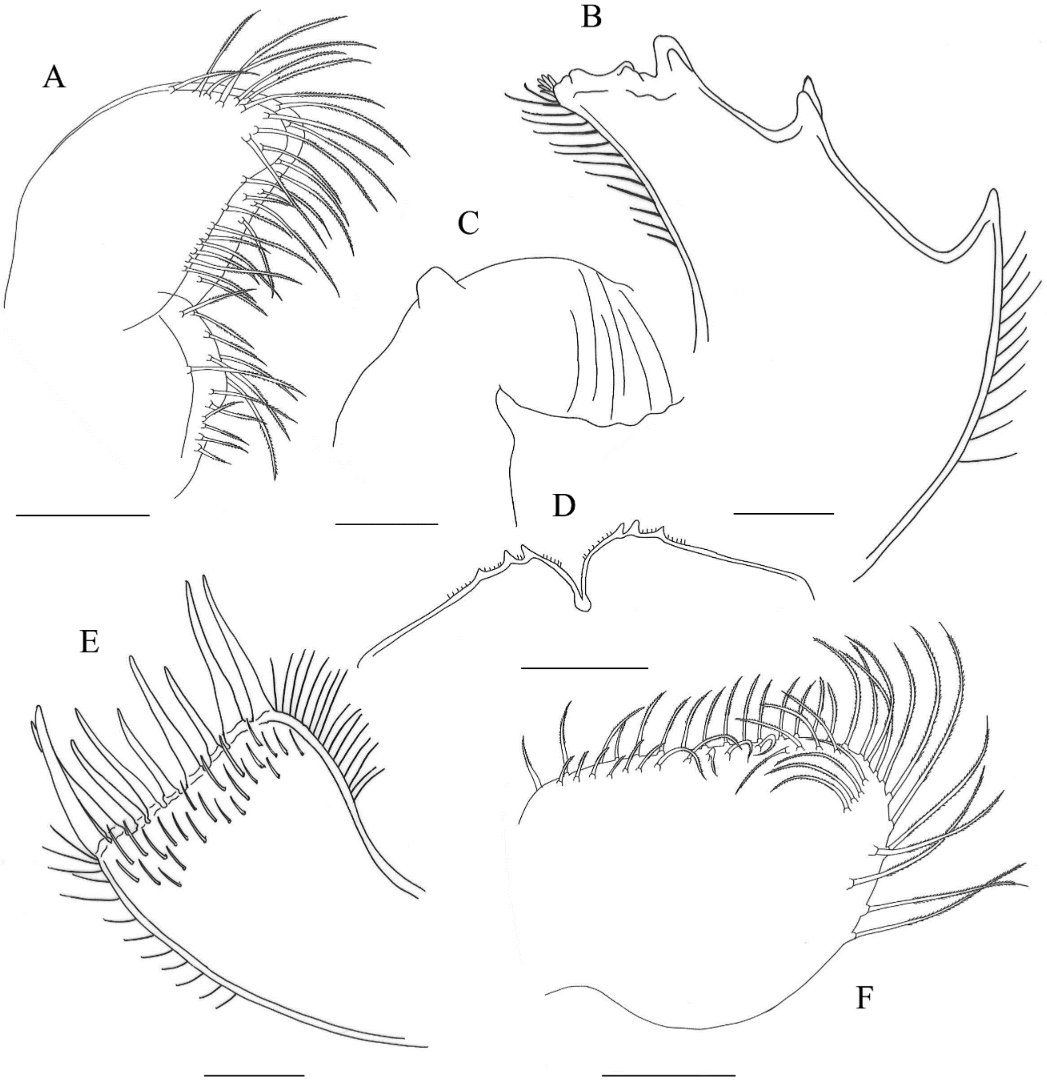

S8 Fig. *Cantellius* cf. *eusponulosum*. Line drawing showing. A, Maxilla. B, Mandible. C, Basi-dorsal point of penis. D, Labrum. E, Maxillule. F, Mandibulatory palp. Scale bars: A, E=0.1 mm, B~D, F=0.2 mm.

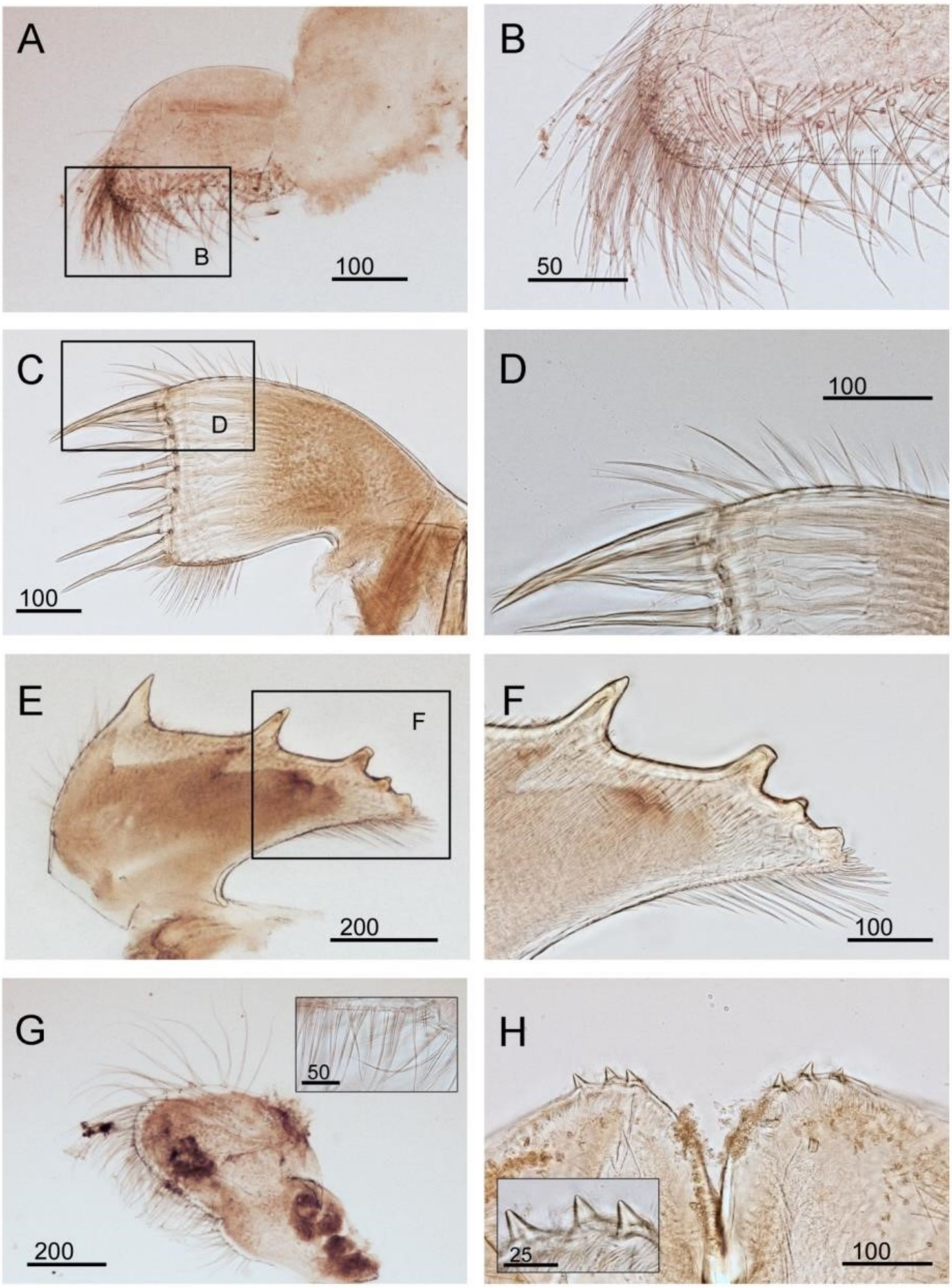

S9 Fig. ***Cantellius*** **cf.** ***eusponulosum*****.** Light microscopy showing. A. Maxilla, B. External lobe of maxilla, C. Maxillule, D. Cutting margin and external margin of maxillule showing simple type setae, E. Mandible, F. Second, third, fourth and fifth teeth of mandible, G. Mandibulatory palp, H. Labrum. Scale bars in µm.

Cirrus I with unequal rami, anterior ramus long, slender, with 11-segments,

posterior ramus 9-segmented (S10A Fig), bearing serrulate setae (S10A Fig). Cirrus II (S10B Fig) anterior ramus with12-segments, slightly longer than posterior ramus (7-segmented), bearing serrulate setae (S10B Fig). Cirrus III anterior ramus longer than posterior ramus, 14- and 10-segmented respectively (S10C Fig), bearing serrulate setae (S10D Fig), with small sharp teeth on base of each segment (S10D Fig). Cirri IV-VI long, slender, rami similar in length (S10E-S10G Figs), bearing serrulate setae. Cirrus IV with anterior ramus 33-segmented, posterior ramus 27-segmented, Cirrus V (anterior 30-segmented, posterior 35-segmented), Cirrus VI (anterior 30-segmented, posterior 30-segmented). Each intermediate segment of ramus of Cirrus IV-VI with 2 pairs of long serrulate setae and 2 pairs of short simple setae. Penis annulated, with scattered short simple-type setae (S10H Fig). Pedicel with sharp basidorsal point (S8C and S10H Figs).

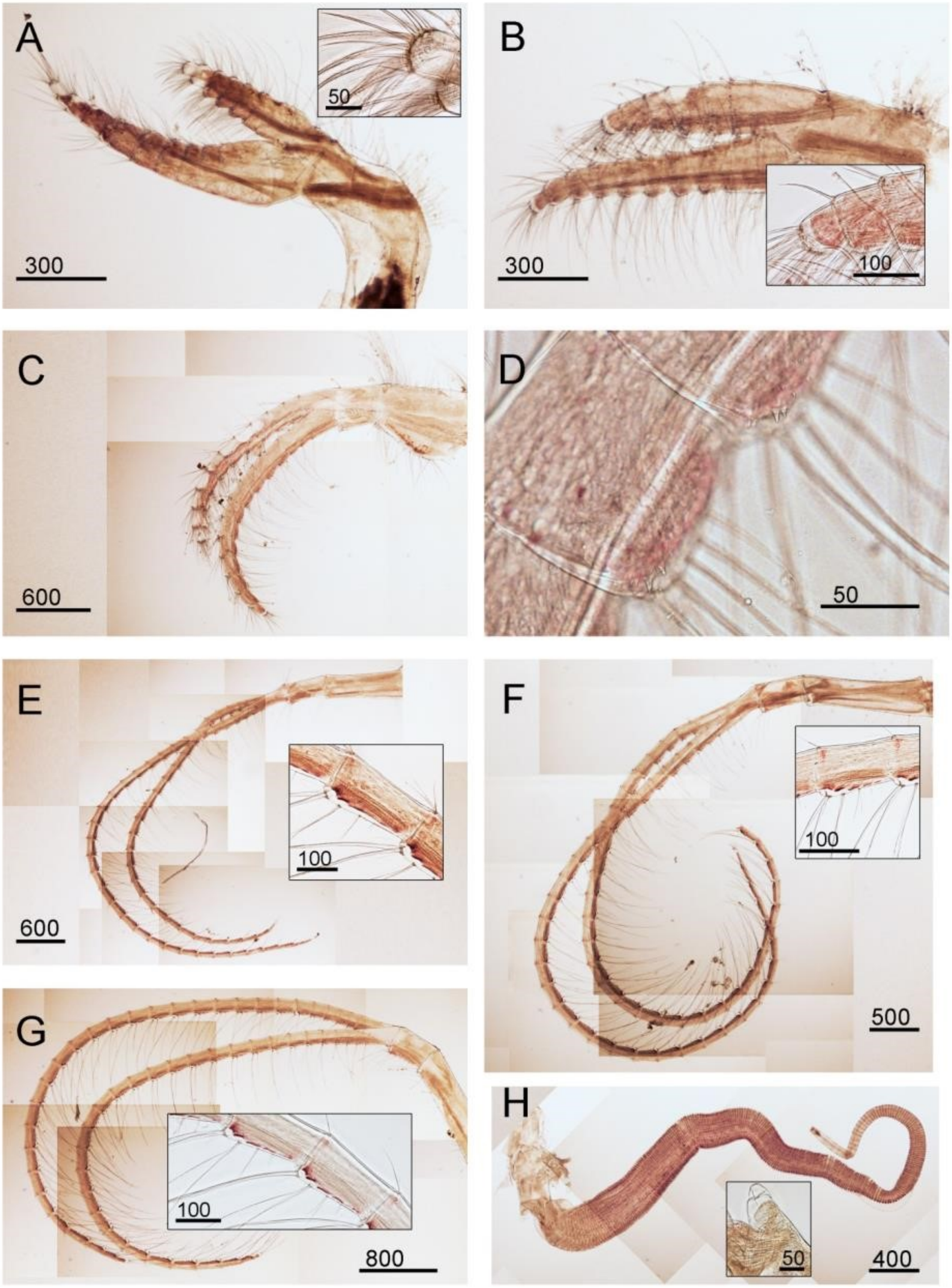

S10 Fig. *Cantellius* cf. *eusponulosum*. Light microscopy on A. Cirrus I, B. Cirrus II, C. Cirrus III, D. Posterior ramus of cirrus III showing the small spines, E. Cirrus IV, F. Cirrus V, G. Cirrus VI, inserts in E-G showing intermediate segments, H. Penis. Insert showing basi-dorsal point. Scale bars in µm.

Habitat. On host coral *Alveopora japonica*

Distribution. At present only recorded in Jeju, Korea.

Remarks. The morphology of scutum and tergum of this species is similar to *Cantellius euspinulosum*. In the present study, the rostral tooth of our specimen is unapparent, which is differing from Hiro [1] in which the specimens had an obvious rostral tooth. *C. euspinulosum* illustrated in Darwin [14] has a small rostral tooth. The size of rostral tooth in *C. euspinulosum* may be a variable morphological character.

Subfamily Megatrematinae Holthuis, 1982

Genus *Pyrgomina* Baluk and Radwanski, 1967

Pyrgomina oulastreae Utinomi, 1962

S1E-S1H and S2I-S2L and S11-S13 Figs

Pyrgoma anglicum. —Hiro, 1935: 9, in part, not fig 4. [18]

Creusia spinulosa forma *quarta*. —Utinomi,1949b: 35, fig 6. [26]

Pyrgoma oulastreae Utinomi, 1962: 227, figs 6-8. [27] —Harada, 1991: 196 (infomration on type specimens). [28]

Boscia oulastrea. —Ross and Newman, 1973: 164. [29] —Foster, 1982: Table 5, fig.
7D, Pl 1A. [7]

Megatrema oulastreae. —Kim 2011: 107, fig 58. [24]

Pyrgomina oulastreae. —Ross and Pitombo, 2002: 61. [30]

Materials examined. CEL-JJ-03, 14 specimens (BD 1.10 – 6.71 mm), 33°13.917' N 126°35.800' E, Seop Seom, Jeju Island, Korea, 6 August 2016, coll. B.K.K. Chan, on host coral *Oulastrea crispata*. CEL-JJ-04, 5 specimens (BD 5.42 – 6.09 mm), 33° 13.917' N 126°35.800' E, Seop Seom, Jeju Island, Korea, 6 August 2016, coll. B.K.K. Chan, on host coral *Psammocora* sp. CEL-JJ-05, 9 specimens (BD 4.29 – 8.35 mm), same data as CEL-JJ-04. CEL-JJ-06, 43 specimens (BD 2.32 – 5.30 mm), same data as CEL-JJ-03. CEL-JJ-10, 10 specimens (BD 2.82 – 6.02 mm), same data as CEL-JJ-04. CEL-JJ-11, 107 specimens (BD 1.58 – 6.31 mm), same data as CEL-JJ-04. CEL-JJ-21, 6 specimens (BD 3.87 – 6.29 mm), Seop Seom (NE), Jeju Island, Korea, 7 August 2016, coll. B.K.K. Chan, on host coral *Oulastrea crispata*. CEL-JJ-40, 11 specimens (BD 7.23 – 9.36 mm), 33°13.115' N, 126°30.850' E, Beom Seom, Jeju Island, Korea, 10 August 2016, coll. B.K.K. Chan, on host coral *Psammocora* sp. CEL-JJ-42, 4 specimens (BD 2.15 – 6.23 mm), Beom Seom, Jeju Island, Korea, 10 August 2016, coll. B.K.K. Chan, on host coral *Oulastrea crispata* CEL-JJ-44, 26 specimens (BD 2.38 – 5.36 mm), 33° 13.345' N 126°30.753' E, Beom Seom, Jeju Island, Korea, 11 August 2016, coll. B.K.K. Chan, on host coral *Oulastrea crispata*.

Diagnosis. Shell single plated, conical and solid, shell with 30-36 longitudinal

ribs. Scutum and tergum separate and balanoid type. Internal side of scutum with well-developed lateral depressor muscle crests.

Description. Shell conical, single-plated complete shell. External surface covered by coral tissue (S1E-S1H and S2I Figs). Base of shell with approximately 35 longitudinal septa radiating from rim of sheath to external shell surface, septa margin serrated (S2J and S11A Figs). From basal view, sheath with two lineation referred as pseudoalae[1] (S11A Fig) [5].

Orifice circular, about 1/4 length of rostro-carinal diameter. Scutum and tergum balanoid and separated (S2K and S2L Figs). Scutum triangular, width approximately equal to height, occludent margin straight with 11 teeth, rostral tooth and adductor plate absent (S11B Fig). Internal view with an oval-shaped adductor and depressor muscle scar (S2L and S11C Figs). External surface with horizontal striations (S11B Fig). Tergum triangular. Spur wide and blunt, width of spur about half wide of tergum (S2L and S11D and S11E Figs). Basi-scutal angle present on scutal margin, at about 100° (S11E Fig). External surface with a medial furrow, extending from basal margin towards apex, width of furrow increasing gradually from apex to base (S11D Fig). External surface with horizontal striations (S11D Fig).

---

[1] Pseudoalae is the position of the sheath where the junction of scutum and tergum attach on the basal margin of the sheath. This is the point of pivotal support of opercular plates during early ontogenic development [31].

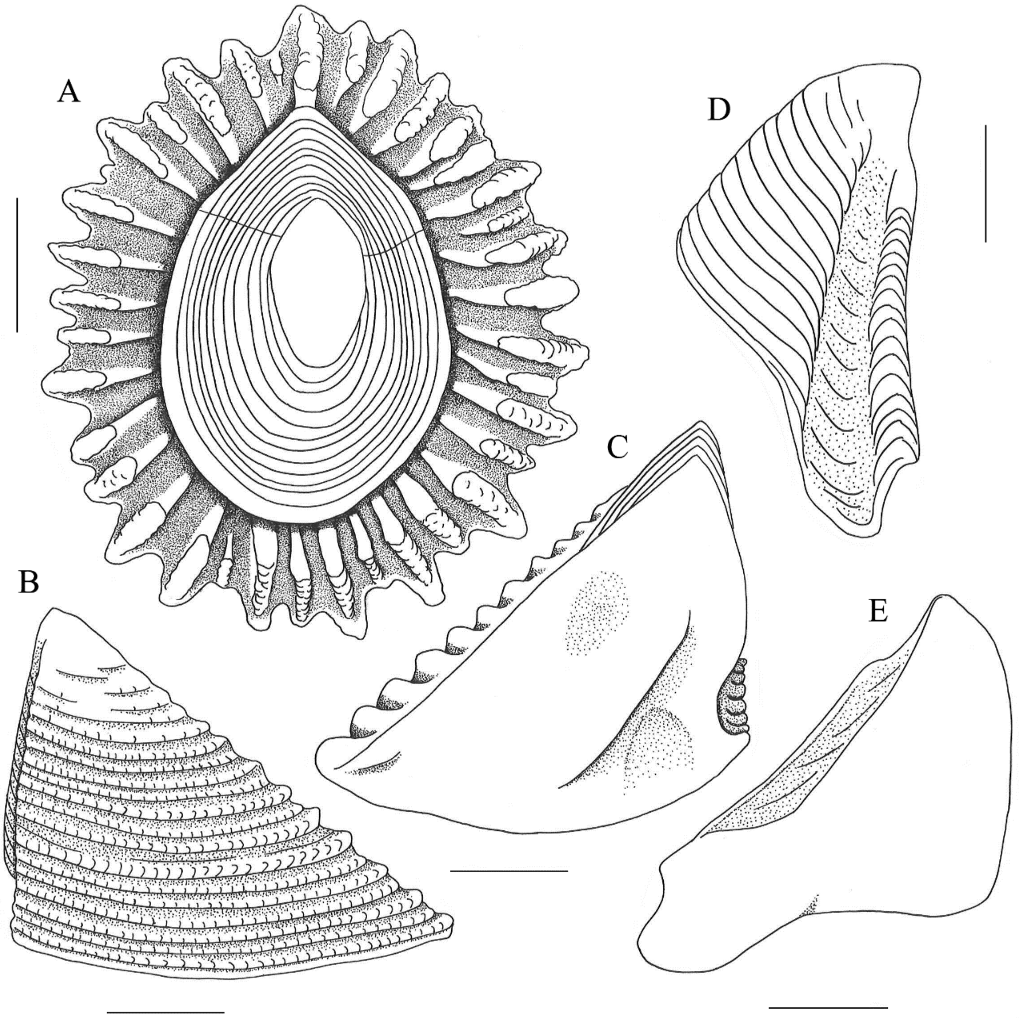

S11 Fig. *Pyrgomina oulastreae*. Line drawing showing. A, Internal view of shell. B, External view of scutum. C, Internal view of scutum. D, External view of tergum. E, Internal view of tergum. Scale bars: A=1 mm, B, C=0.4 mm, D, E=0.25 mm.

Maxilla ovate (S12A Fig), with serrulate setae on margin. Maxillule cutting edge straight with a shallow notch, 2 large setae above notch and 5 setae below notch (S12F Fig). Anterior and posterior margins with simple-type setae. Mandible with 5 teeth, excluding inferior angle (S12E Fig). Second to fourth teeth bidentate, first 3 teeth occupied 3/5 length of cutting edge (S12E Fig). Lateral surface, lower margin and cutting edge of mandible bearing simple-type setae. Lower margin short, inferior angle blunt with simple-type setae. Mandibular palp elongated (S12B Fig), bearing serrulate setae distally and on interior margin. Labrum bilobed, lobes separated by a V-shaped notch, 3 sharp teeth on each side of notch (S12D Fig).

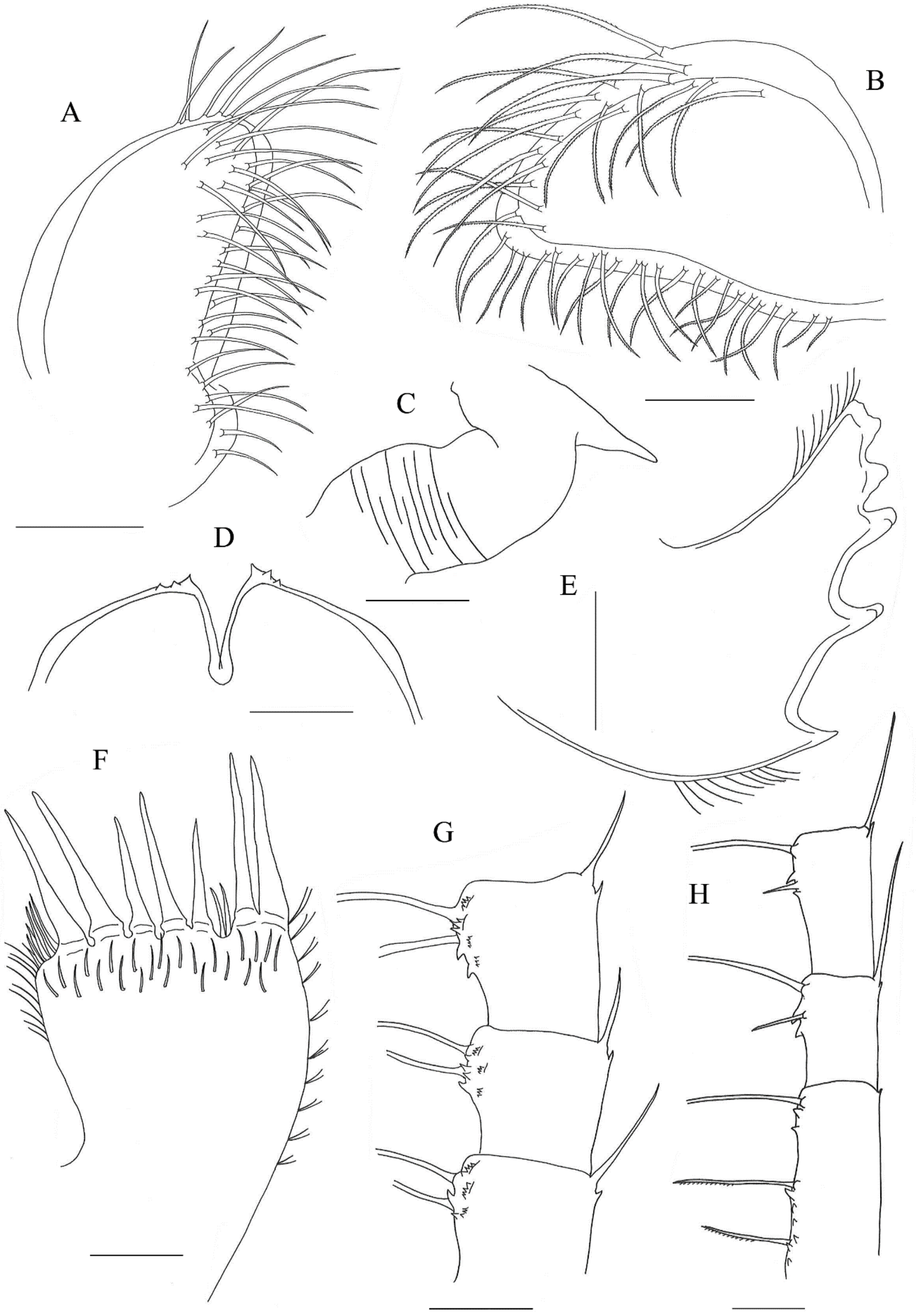

S12 Fig. ***Pyrgomina oulastreae.*** Line drawing showing. A, Maxilla. B, Mandibulatory palp. C, Basi-dorsal point of penis. D, Labrum. E, Mandible. F, Maxillule. G, Intermediate segment of cirrus III, showing the spines. H, Intermediate segment of cirrus IV, showing the spines. Scale bars: A, B, D, E=0.1 mm, C=0.2 mm, F, G, H=0.05 mm.

Cirrus I with unequal rami, anterior ramus long, slender, with 13-segments, posterior ramus 8-segmented (S13A Fig), bearing serrulate setae. Cirrus II (S13B Fig) anterior ramus with 9 segments, slightly longer than posterior ramus (6-segmented), bearing serrulate setae. Cirrus III anterior ramus longer than posterior ramus, 13- and 11-segmented respectively (S13C Fig), bearing serrulate setae. Cirri IV-VI long, slender, rami similar in length (S13D and S13E Figs) , bearing serrulate setae. Cirrus IV with anterior ramus 29-segmented, posterior ramus 29-segmented, Cirrus V (anterior 30-segmented, posterior 35-segmented), Each intermediate segment of ramus of Cirrus IV-VI with 1 pairs of long serrulate setae and 1 pairs of short simple setae. Penis annulated, with scattered short simple-type setae. Pedicel with small basidorsal point (S12C Fig).

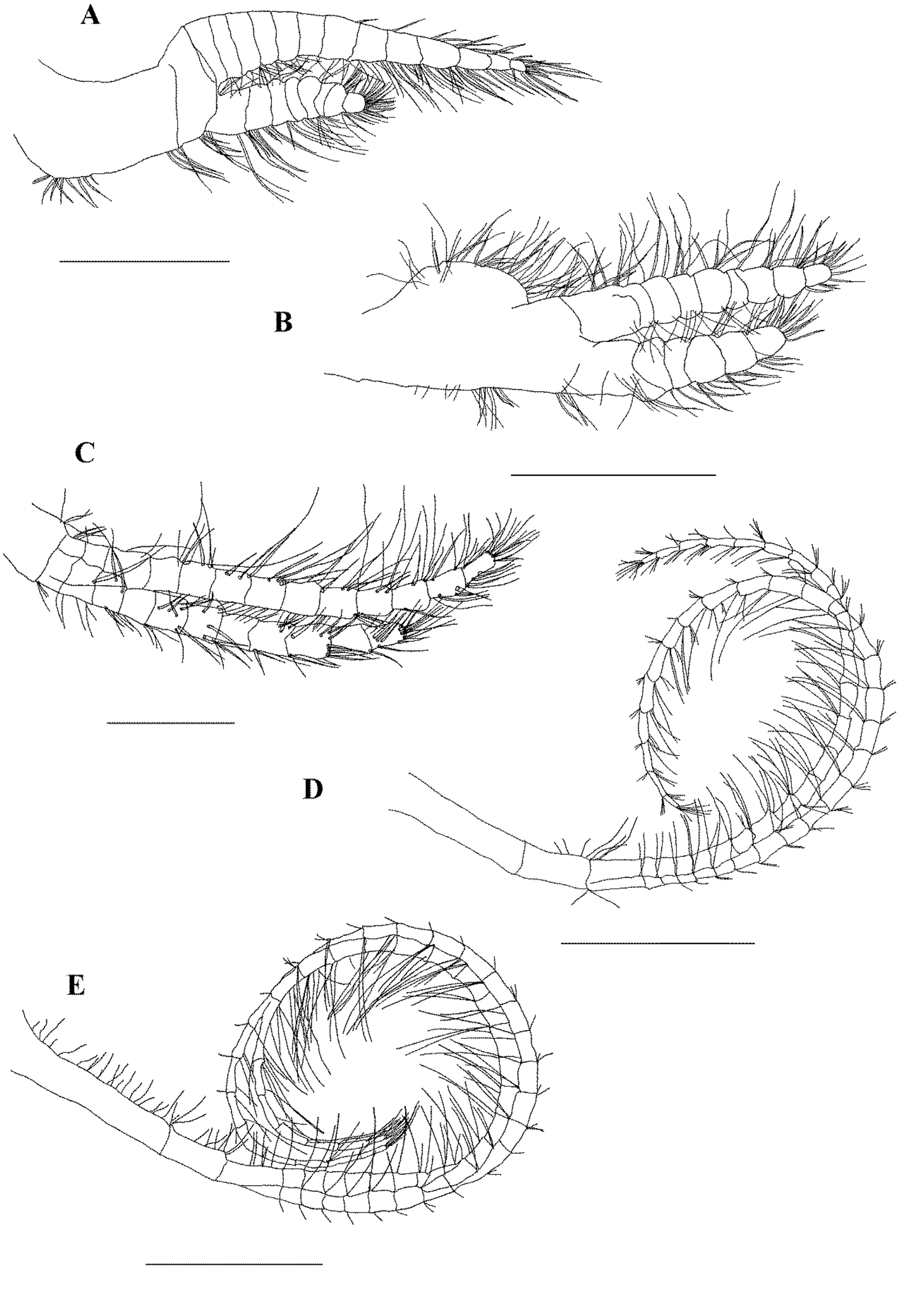

S13 Fig. ***Pyrgomina oulastreae*****.** Line drawing showing. A, Cirrus I. B, Cirrus II. C, Cirrus III. D, Cirrus IV. E, Cirrus V. Scale bars: A–E=1.1 mm.

Habitat. On host coral *Psammocora* spp.*, Oulastrea crispata* and *Dichopsammia granulosa* [24].

Distribution. Japan and Korea

Remarks. Ross and Newman [5] revised the family Pyrgomatidae, in which this family is divided into three subfamilies including Pyrgomatinae (4 plate or united shells, separated or fused scutum and tergum), Ceratoconchinae (4 plate shells, separated scutum and tergum) and Bosciinae (fused shell and with separated balanoid shaped scutum and tergum). The subfamily Bosciinae composed of one genus *Boscia*. In Ross and Newman [5], they concluded the name *Megatrema* is synonym to *Boscia*. However, Holthuis [31] found that the genus *Boscia* is pre-occupied. Holthuis explained *Megatrema* is the correct name to replace *Boscia*. Since *Boscia* is replaced, the subfamily name changed from Bosciinae to Megatrematinae.

Ross and Pitombo [30] conducted a revision in the subfamily Megatrematinae. In this revision, they place *Megatrema oulastreae* into the genus *Pyrgomina*.

References

1. Hiro F. Studies on the animals inhabiting reef corals. II. Cirripedes of the genera *Creusia* and *Pyrgoma*. Palao Tropical Biological Station Studies 3. 1938;391–417.
2. Kolosváry G. A study of Cirripedes associated with corals in the collection of the Hungarian National Museum, Budapest. Proceedings of the Zoological Society of London. 1947a;117:425–428.
3. Kolosváry G. New data of Cirripeds associated with corals. Journal of Natural History Series11. 1947b;14:358–368.
4. Utinomi H. Studies on the cirripedia of Japan. I. Classification and differentiation of species. Seibutsu 4. 1949a;62-70.
5. Ross A, Newman WA. Revision of the coral inhabiting barnacles (Cirripedia: Balanidae). Transactions of the San Diego Society of Natural History. 1973;17(12):137–174.
6. Newman W, Ross A. Revision of the Balanomorph barnacles; including a catalog of the species. Memoirs of the San Diego Society of Natural History. 1976;9:1–108.
7. Foster BA. Shallow water barnacles from Hong Kong. Proceedings of the First International Marine Biological Workshop: The Marine Flora and Fauna of Hong Kong and southern China, Hong Kong, 1980. B. Morton (Ed). 1982:207–232.
8. Ogawa K, Matsuzaki K. Revision of the coral-inhabiting barnacles in Japan—preliminary note. Nanki-Seibutu, Nanki Biological

Society.1990;32:73-79.
9. Galkin SV. The system of coral-inhabiting barnaclrs (Cirripedia, Balanomorpha). Zoologichesky Zhurnal. 1986b;65(9):1285–1295.
10. Ogawa K. An essay on host specificity, systematic taxonomy, and evolution of the coral barnacles. Biogeographical Society of Japan. 1992;47(10):87–101.
11. Asami K, Yamaguchi T. Distribution of living and fossil coral barnacles (Cirripedia; Pyrgomatidae) in Japan. Sessile Organisms. 1997;14(1):9–16.
12. Ogawa K, Pillay RM, Kawasaki H. Coral-Inhabiting barnacles (Cirripedia; Pyrgomatidae) from Albion, west coast of Republic of Mauritius. Bulletin of the Biogeographical Society of Japan. 1998;53:1–21.
13. Chan BKK, Chen YY, Achituv Y. Crustacean Fauna of Taiwan: Barnacles Volume II: Cirripedia: Thoracica: Pyrgomatidae. Biodiversity Research Center, Academia Sinica Press. 2013a:367.
14. Darwin C. A monograph on the sub-class Cirripedia with figures of all the species. The Balanidae, the Verrucidae, etc. Royal Society, London. 1854:684.
15. Gruvel A. Monographie des Cirripedes ou Thecostraces. Masson et Cie, Paris. 1905:427.
16. Broch, H. Papers from Dr. Th. Mortensen's Pacific Expedition 1914-16. LVI. Indomalayan Cirripedia. Videnskabelige meddelelser fra den Naturhistoriske forening Kjobenhavn.1931;91:1–146.
17. Nilsson-Cantell CA. Cirripedes from the Indian Ocean in the collection of the Indian Museum, Calcutta. Memoirs of the Indian Museum. 1938;13:1–81.
18. Hiro F. A study of cirripeds associated with corals occurring in Tanabe Bay. Records of Oceanographic Works in Japan. 1935;7:45–72.
19. Jones DS, Hewitt MA, Sampey A. A checklist of the Cirripedia of the South China Sea. Raffles Bulletin of Zoology. 2000:233–307.
20. Achituv Y, Newman WA. The barnacles of *Astreopora* (Cirripedia, Pyrgomatini/Scleractinia, Acroporidae): Organization plans, host specificity, species-richness and geographic range. Journal of Natural History. 2002;36:391–406.
21. Anderson DT. Structure, function and phylogeny of coral-inhabiting barnacles (Cirripedia, Balanoidea). Aoological journal of the Linnean Society. 1992;106(4):277–339.
22. Paulay G, Ross A. An annotated checklist of the shallow water Cirripedia of Guam. Micronesica. 2003;35–36:303–314.
23. Ogawa K, Tachikawa H. Coral-Inhabiting barnacles from the Ogasawara

Islands. Bulletin of the Biogeographical Society of Japan.2009; 64:89–96.

24. Kim IH. Barnacles. National Institute of Biological Resources, Ministry of Environment, South Korea. vertebrate Fauna of Korea. 2011;21(6):105–108.
25. Poltarukha OP, Dautova TN. Barnacles (Cirripedia, Thoracica) of Nhatrang Bay. Benthic fauna of the Bay of Nhatrang Southern Vietnam. KMK Scientific Press, Moscow. 2007:89–123.
26. Utinomi H. Studies on the cirripedia fauna of Japan. VI. Cirripeds from Kyusyu and Ryukyu Islands. Publications of the Seto Marine Biological Laboratory. 1949b;1:19–37.
27. Utinomi H. Studies on the Cirripedian fauna of Japan. VIII. Thoracic cirripeds from western Kyushu. Publications of the Seto Marine Biological Laboratory. 1962;10:211–239.
28. Harada E. Inventory of zoological type specimens in the Museum of the Seto Marine Biological Laboratory. Publications of the Seto Marine Biological Laboratory. 1991;35(1-3):171–233.
29. Ross A, Newman WA. Revision of the coral inhabiting barnacles (Cirripedia: Balanidae). Trans San Diego Society of Natural History. 1973;17:137–174.
30. Ross A, Pitombo FB. Notes on the coral-inhabiting Megatrematinae and the description of a new tribe, new genus and three new species (Cirripedia: Sessilia: Pyrgomatidae). Sessile Organisms. 2002;14:9–16.
31. Holthuis LB. The nomenclature of some coral-inhabiting barnacles of the family Pyrgomatidae (Cirripedia, Balanomorpha). Crustaceana. 1982;43(3):316–320.
