# Supplementary figures and images for: Living with marginal coral communities: Diversity and host-specificity in coral-associated barnacles in the northern coral distribution limit of the East China Sea
Source: PLoS One. 2018 May 1;13(5):e0196309. doi: 10.1371/journal.pone.0196309 (PMC5929504; doi:10.1371/journal.pone.0196309)

# Maximum likelihood analysis of 12S

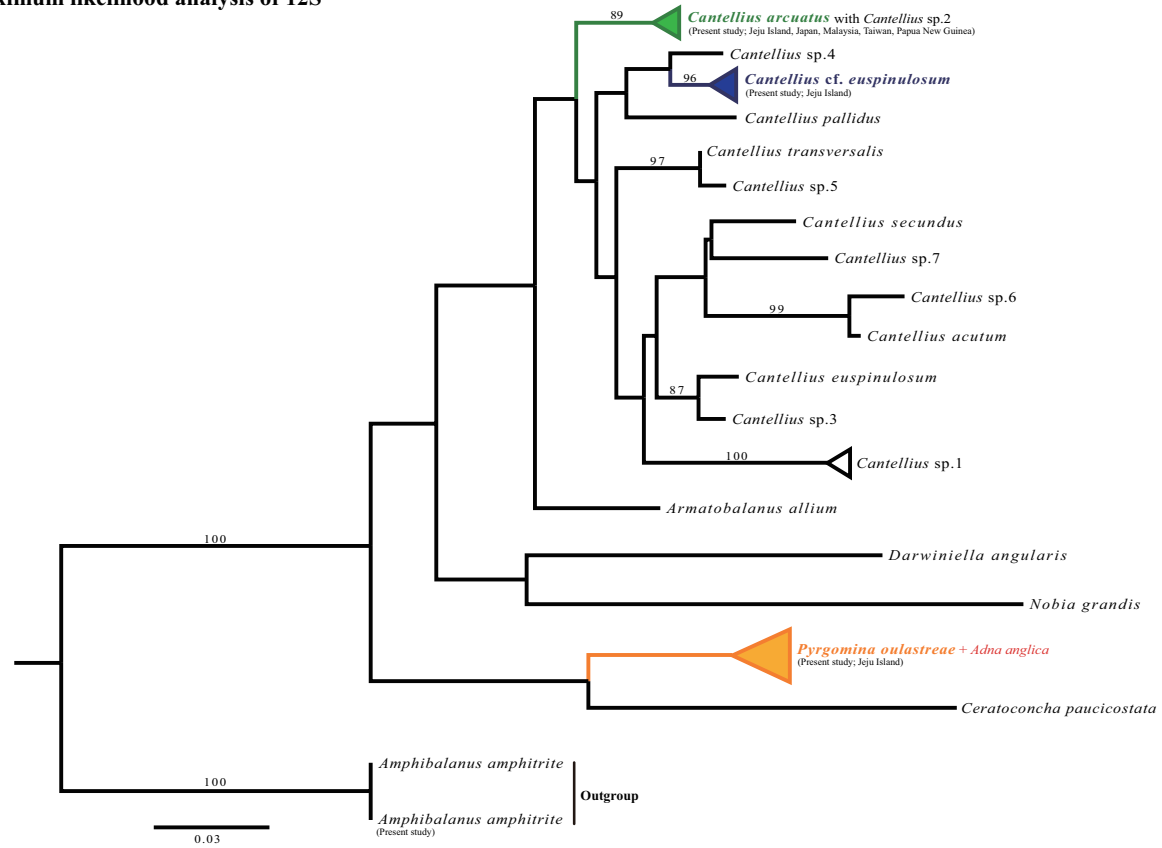

Maximum likelihood analysis of COI

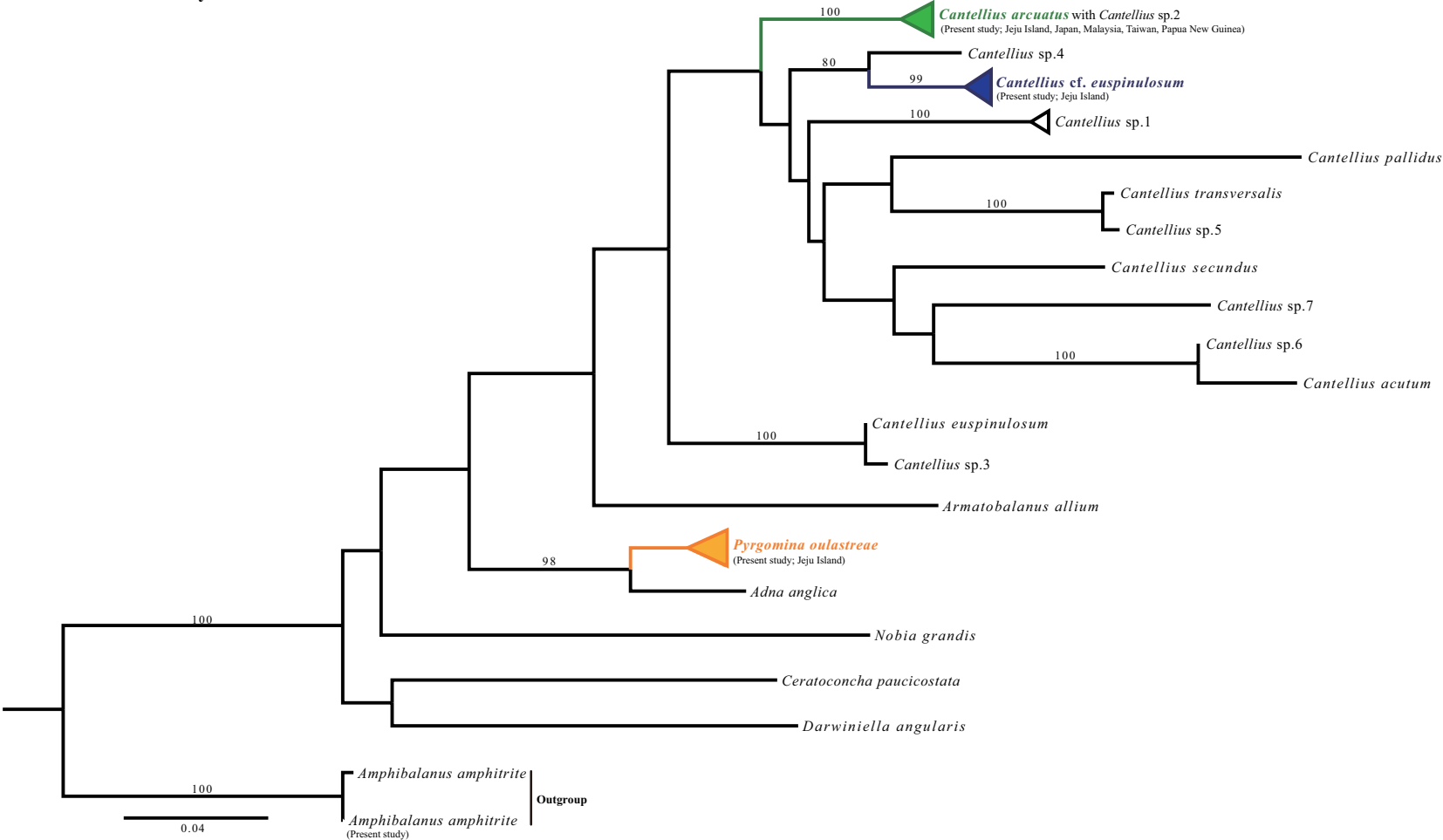

Supplement: S2 File — (PDF) [file pone.0196309.s002.pdf]
