# Supplementary material for: Living with marginal coral communities: Diversity and host-specificity in coral-associated barnacles in the northern coral distribution limit of the East China Sea
Source: PLoS One. 2018 May 1;13(5):e0196309. doi: 10.1371/journal.pone.0196309 (PMC5929504; doi:10.1371/journal.pone.0196309)
Supplement: S1 Table — (PDF) [file pone.0196309.s003.pdf]

S1 Table. Information of collection sites and Genebank no. for the sequences used for the phylogenetic analysis.

Voucher no.	Species name	Location	COI References	Accession no. COI	12S References	Accession no. 12S
UF 26330	*Adna anglica*	Spain on coral *Oculina patagonica*	Malay and Michonneau 2014	HG970570	Malay and Michonneau 2014	HG970509
UF26338	*Adna anglica*	Spain on coral *Oculina patagonica*	Malay and Michonneau 2014	HG970569	Malay and Michonneau 2014	HG970508
UF26329	*Adna anglica*	Spain on coral *Oculina patagonica*	Malay and Michonneau 2014	HG970568	Malay and Michonneau 2014	HG970507
UF8634	*Cantellius* sp.1	Philippines on coral *Pachyseris rugosa*	Malay and Michonneau 2014	HG970542	Malay and Michonneau 2014	HG970484
UF8636	*Cantellius* sp.1	Philippines on coral *Pachyseris rugosa*	Malay and Michonneau 2014	HG970543	Malay and Michonneau 2014	HG970485

UF8670	*Cantellius* sp.2	Philippines on coral *Montipora* sp.	Malay and Michonneau 2014	HG970552	Malay and Michonneau 2014	HG970494
UF8638	*Cantellius* sp.3	Philippines on coral *Porites* sp.	Malay and Michonneau 2014	HG970558	Malay and Michonneau 2014	HG970500
UF8663	*Cantellius* sp.3	Philippines on coral *Porites* sp.	Malay and Michonneau 2014	HG970551	Malay and Michonneau 2014	HG970493
UF8664	*Cantellius* sp.4	Philippines on coral *Montipora* sp.	Malay and Michonneau 2014	HG970555	Malay and Michonneau 2014	HG970497
UF6541	*Cantellius* sp.5	Philippines on coral *Acropora* sp.	Malay and Michonneau 2014	HG970571	Malay and Michonneau 2014	HG970510
UF8676	*Cantellius* sp.6	Philippines on coral *Acropora* sp.	Malay and Michonneau 2014	HG970553	Malay and Michonneau 2014	HG970495

UF8651	*Cantellius* sp.7	Philippines on coral *Acropora* sp.	Malay and Michonneau 2014	HG970554	Malay and Michonneau 2014	HG970496
UF13228	*Ceratoconcha paucicostata*	Florida on coral *Madracis* sp.	Malay and Michonneau 2014	HG970563	Malay and Michonneau 2014	HG970504
CEL-GI164-1	*Cantellius acutum*	Green Island, Taiwan on coral *Acropora lutkeni*	Present study	MG878744	L.M. Tsang et al. 2014	KF776142
CEL-KT16-1	*Cantellius euspinulosum*	Kenting, Taiwan on coral *Porites* sp.	Present study	MG878750	L.M. Tsang et al. 2014	KF776147
CEL-KT35-3	*Cantellius* sp.1	Kenting Taiwan, on coral *Pachyseris speciosa*	Present study	MG878745	L.M. Tsang et al. 2014	KF776150
CEL-GI170-1	*Cantellius pallidus*	Green Island, Taiwan on coral *Pocillopora* sp.	Present study	MG878746	L.M. Tsang et al. 2014	KF776151

CEL-TI6-3	*Cantellius secundus*	Kuei Shan Island, Taiwan on coral *Acropora muricata*	Present study	MG878747	L.M. Tsang et al. 2014	KF776153
CEL-SU46-1	*Cantellius transversalis*	Suao, Taiwan on coral *Acropora elseyi*	Y.R. Cheng et al. (Unpublished)	MF576463	L.M. Tsang et al. 2014	KF776154
CEL-TI1-4	*Darwinella angularis*	Kuei Shan Island, Taiwan on coral *Cyphastrea chalcidicum*	Present study	MG878748	L.M. Tsang et al. 2014	KF776155
CEL-KT100-1	*Nobia grandis*	Kenting, Taiwan on coral *Galaxea fascicularis*	Present study	MG878749	L.M. Tsang et al. 2014	KF776166
CEL-GI175-1	*Armatobalanus allium*	Green Island, Taiwan on coral *Porties* sp.	Present study	MG878743	L.M. Tsang et al. 2014	KF776138
CEL-JJ01-02	*Cantellius arcuatus*	Site 316, Jeju Island, Korea	Present study	MG878629	Present study	MG878752

CEL-JJ01-03	*Cantellius arcuatus*	Site 316, Jeju Island, Korea	Present study	MG878630	Present study	MG878753
CEL-JJ01-04	*Cantellius arcuatus*	Site 316, Jeju Island, Korea	Present study	MG878631	Present study	MG878754
CEL-JJ02-02	*Cantellius arcuatus*	Site 316, Jeju Island, Korea	Present study	MG878632	Present study	MG878755
CEL-JJ02-03	*Cantellius arcuatus*	Site 316, Jeju Island, Korea	Present study	MG878633	Present study	MG878756
CEL-JJ02-04	*Cantellius arcuatus*	Site 316, Jeju Island, Korea	Present study	MG878634	Present study	MG878757
CEL-JJ13-02	*Cantellius arcuatus*	Site 316, Jeju Island, Korea	Present study	MG878664	Present study	MG878779

CEL-JJ13-03	*Cantellius arcuatus*	Site 316, Jeju Island, Korea	Present study	MG878665	Present study	MG878780
CEL-JJ13-05	*Cantellius arcuatus*	Site 316, Jeju Island, Korea	Present study	MG878666	Present study	MG878781
CEL-JJ14-02	*Cantellius arcuatus*	Site 316, Jeju Island, Korea	Present study	MG878667	Present study	MG878782
CEL-JJ14-03	*Cantellius arcuatus*	Site 316, Jeju Island, Korea	Present study	MG878668	Present study	MG878783
CEL-JJ14-04	*Cantellius arcuatus*	Site 316, Jeju Island, Korea	Present study	MG878669	Present study	MG878784
CEL-JJ15-01	*Cantellius arcuatus*	Site 316, Jeju Island, Korea	Present study	MG878670	Present study	MG878785

CEL-JJ15-02	*Cantellius arcuatus*	Site 316, Jeju Island, Korea	Present study	MG878671	Present study	MG878786
CEL-JJ15-05	*Cantellius arcuatus*	Site 316, Jeju Island, Korea	Present study	MG878672	Present study	MG878787
CEL-JJ16-02	*Cantellius arcuatus*	1st Dive, Jeju Island, Korea	Present study	MG878673	Present study	MG878788
CEL-JJ16-03	*Cantellius arcuatus*	1st Dive, Jeju Island, Korea	Present study	MG878674	Present study	MG878789
CEL-JJ16-04	*Cantellius arcuatus*	1st Dive, Jeju Island, Korea	Present study	MG878675	Present study	MG878790
CEL-JJ17-02	*Cantellius arcuatus*	Site 318, Jeju Island, Korea	Present study	MG878676	Present study	MG878791

CEL-JJ17-03	*Cantellius arcuatus*	Site 318, Jeju Island, Korea	Present study	MG878677	Present study	MG878792
CEL-JJ17-04	*Cantellius arcuatus*	Site 318, Jeju Island, Korea	Present study	MG878678	Present study	MG878793
CEL-JJ18-02	*Cantellius arcuatus*	1st Dive, Jeju Island, Korea	Present study	MG878679	Present study	MG878794
CEL-JJ18-03	*Cantellius arcuatus*	1st Dive, Jeju Island, Korea	Present study	MG878680	Present study	MG878795
CEL-JJ18-04	*Cantellius arcuatus*	1st Dive, Jeju Island, Korea	Present study	MG878681	Present study	MG878796
CEL-JJ19-02	*Cantellius arcuatus*	Big Rock, 1st Dive, Jeju Island, Korea	Present study	MG878682	Present study	MG878797

CEL-JJ19-03	*Cantellius arcuatus*	Big Rock, 1st Dive, Jeju Island, Korea	Present study	MG878683	Present study	MG878798
CEL-JJ19-04	*Cantellius arcuatus*	Big Rock, 1st Dive, Jeju Island, Korea	Present study	MG878684	Present study	MG878799
CEL-JJ20-02	*Cantellius arcuatus*	1st Dive, Jeju Island, Korea	Present study	MG878685	Present study	MG878800
CEL-JJ20-03	*Cantellius arcuatus*	1st Dive, Jeju Island, Korea	Present study	MG878686	Present study	MG878801
CEL-JJ20-04	*Cantellius arcuatus*	1st Dive, Jeju Island, Korea	Present study	MG878687	Present study	MG878802
CEL-JJ23-02	*Cantellius arcuatus*	Site 318, Jeju Island, Korea	Present study	MG878688	Present study	MG878806

CEL-JJ23-03	*Cantellius arcuatus*	Site 318, Jeju Island, Korea	Present study	MG878689	Present study	MG878807
CEL-JJ23-04	*Cantellius arcuatus*	Site 318, Jeju Island, Korea	Present study	MG878690	Present study	MG878808
CEL-JJ25-02	*Cantellius arcuatus*	Site 318, Jeju Island, Korea	Present study	MG878691	Present study	MG878809
CEL-JJ25-03	*Cantellius arcuatus*	Site 318, Jeju Island, Korea	Present study	MG878692	Present study	MG878810
CEL-JJ25-05	*Cantellius arcuatus*	Site 318, Jeju Island, Korea	Present study	MG878693	Present study	MG878811
CEL-JJ28-03	*Cantellius arcuatus*	Site 321, Jeju Island, Korea	Present study	MG878694	Present study	MG878812

CEL-JJ28-04	*Cantellius arcuatus*	Site 321, Jeju Island, Korea	Present study	MG878695	Present study	MG878813
CEL-JJ28-05	*Cantellius arcuatus*	Site 321, Jeju Island, Korea	Present study	MG878696	Present study	MG878814
CEL-JJ33-02	*Cantellius arcuatus*	1st Dive, Jeju Island, Korea	Present study	MG878697	Present study	MG878815
CEL-JJ33-03	*Cantellius arcuatus*	1st Dive, Jeju Island, Korea	Present study	MG878698	Present study	MG878816
CEL-JJ33-04	*Cantellius arcuatus*	1st Dive, Jeju Island, Korea	Present study	MG878699	Present study	MG878817
CEL-JJ34-02	*Cantellius arcuatus*	Site 318 (2nd), Jeju Island, Korea	Present study	MG878700	Present study	MG878818

CEL-JJ34-03	*Cantellius arcuatus*	Site 318 (2nd), Jeju Island, Korea	Present study	MG878701	Present study	MG878819
CEL-JJ34-04	*Cantellius arcuatus*	Site 318 (2nd), Jeju Island, Korea	Present study	MG878702	Present study	MG878820
CEL-JJ35-01	*Cantellius arcuatus*	Site 323, Beom Sum, Jeju Island, Korea	Present study	MG878703	Present study	MG878821
CEL-JJ36-02	*Cantellius arcuatus*	1st Dive, Beom Sum, Jeju Island, Korea	Present study	MG878704	Present study	MG878822
CEL-JJ36-03	*Cantellius arcuatus*	1st Dive, Beom Sum, Jeju Island, Korea	Present study	MG878705	Present study	MG878823
CEL-JJ36-04	*Cantellius arcuatus*	1st Dive, Beom Sum, Jeju Island, Korea	Present study	MG878706	Present study	MG878824

CEL-JJ38-02	*Cantellius arcuatus*	1st Dive, Beom Sum, Jeju Island, Korea	Present study	MG878716	Present study	MG878828
CEL-JJ38-03	*Cantellius arcuatus*	1st Dive, Beom Sum, Jeju Island, Korea	Present study	MG878717	Present study	MG878829
CEL-JJ38-04	*Cantellius arcuatus*	1st Dive, Beom Sum, Jeju Island, Korea	Present study	MG878718	Present study	MG878830
CEL-JJ41-03	*Cantellius arcuatus*	1st Dive, Beom Sum, Jeju Island, Korea	Present study	MG878719	Present study	MG878836
CEL-JJ41-04	*Cantellius arcuatus*	1st Dive, Beom Sum, Jeju Island, Korea	Present study	MG878720	Present study	MG878837
CEL-JJ41-05	*Cantellius arcuatus*	1st Dive, Beom Sum, Jeju Island, Korea	Present study	MG878721	Present study	MG878838

CEL-JJ43-02	*Cantellius arcuatus*	Site 324, Beom Sum, Jeju Island, Korea	Present study	MG878722	Present study	MG878842
CEL-JJ43-03	*Cantellius arcuatus*	Site 324, Beom Sum, Jeju Island, Korea	Present study	MG878723	Present study	MG878843
CEL-JJ43-04	*Cantellius arcuatus*	Site 324, Beom Sum, Jeju Island, Korea	Present study	MG878724	Present study	MG878844
KC-104-02	*Cantellius arcuatus*	Nishidomara, Kochi, Japan on coral *Pavona decussata*	Present study	MG878725	Present study	MG878848
KC-104-03	*Cantellius arcuatus*	Nishidomara, Kochi, Japan on coral *Pavona decussata*	Present study	MG878726	Present study	MG878849
KC-107-02	*Cantellius arcuatus*	Nishidomara, Kochi, Japan on coral *Dipsastraea lizardensis*	Present study	MG878727	Present study	MG878850

KC-107-03	*Cantellius arcuatus*	Nishidomara, Kochi, Japan on coral *Dipsastraea lizardensis*	Present study	MG878728	Present study	MG878851
KC-107-04	*Cantellius arcuatus*	Nishidomara, Kochi, Japan on coral *Dipsastraea lizardensis*	Present study	MG878729	Present study	MG878852
KC-115-01	*Cantellius arcuatus*	Nishidomara, Kochi, Japan on coral *Coscinaraea columna*	Present study	MG878730	Present study	MG878853
KC-115-02	*Cantellius arcuatus*	Nishidomara, Kochi, Japan on coral *Coscinaraea columna*	Present study	MG878731	Present study	MG878854
KC-115-03	*Cantellius arcuatus*	Nishidomara, Kochi, Japan on coral *Coscinaraea columna*	Present study	MG878732	Present study	MG878855
KC-119-09	*Cantellius arcuatus*	Nishidomara, Kochi, Japan on coral *Plesiastrea versipora*	Present study	MG878733	Present study	MG878856

MY-17-02	*Cantellius arcuatus*	Tinggi Island, Malaysia on coral *Montipora undata*	Present study	MG878734	Present study	MG878857
MY-17-03	*Cantellius arcuatus*	Tinggi Island, Malaysia on coral *Montipora undata*	Present study	MG878735	Present study	MG878858
MY-83-01	*Cantellius arcuatus*	Tinggi Island, Malaysia on coral *Montipora undata*	Present study	MG878736	Present study	MG878859
MY-83-02	*Cantellius arcuatus*	Tinggi Island, Malaysia on coral *Montipora undata*	Present study	MG878737	Present study	MG878860
MY-83-03	*Cantellius arcuatus*	Tinggi Island, Malaysia on coral *Montipora undata*	Present study	MG878738	Present study	MG878861
PNG-43-01	*Cantellius arcuatus*	Madang, Papus New Guinea on coral *Psammocora contigua*	Present study	MG878739	Present study	MG878862

PNG-43-04	*Cantellius arcuatus*	Madang, Papus New Guinea on coral *Psammocora contigua*	Present study	MG878740	Present study	MG878863
PNG-43-06	*Cantellius arcuatus*	Madang, Papus New Guinea on coral *Psammocora contigua*	Present study	MG878741	Present study	MG878864
PNG-43-07	*Cantellius arcuatus*	Madang, Papus New Guinea on coral *Psammocora contigua*	Present study	MG878742	Present study	MG878865
CEL- JJ08-02	*Cantellius* cf. *euspinulosum*	Youngsuri, Jeju Island, Korea on *Alveopora japonica*	Present study	MG878707	Present study	MG878770
CEL- JJ08-03	*Cantellius* cf. *euspinulosum*	Youngsuri, Jeju Island, Korea *Alveopora japonica*	Present study	MG878708	Present study	MG878771
CEL- JJ08-04	*Cantellius* cf. *euspinulosum*	Youngsuri, Jeju Island, Korea on *Alveopora japonica*	Present study	MG878709	Present study	MG878772

CEL- JJ37-02	*Cantellius* cf. *euspinulosum*	1st Dive, Beom Sum, Jeju Island, Korea on *Alveopora japonica*	Present study	MG878710	Present study	MG878825
CEL- JJ37-03	*Cantellius* cf. *euspinulosum*	1st Dive, Beom Sum, Jeju Island, Korea on *Alveopora japonica*	Present study	MG878711	Present study	MG878826
CEL- JJ37-04	*Cantellius* cf. *euspinulosum*	1st Dive, Beom Sum, on *Alveopora japonica* Jeju Island, Korea	Present study	MG878712	Present study	MG878827
CEL- JJ39-01	*Cantellius* cf. *euspinulosum*	1st Dive, Beom Sum, on *Alveopora japonica* Jeju Island, Korea	Present study	MG878713	Present study	MG878831
CEL- JJ39-02	*Cantellius* cf. *euspinulosum*	1st Dive, Beom Sum, on *Alveopora japonica* Jeju Island, Korea	Present study	MG878714	Present study	MG878832
CEL- JJ39-03	*Cantellius* cf. *euspinulosum*	1st Dive, Beom Sum, on *Alveopora japonica* Jeju Island, Korea on	Present study	MG878715	Present study	MG878833

CEL-JJ03-02	*Pyrgomina oulastrea*	Site 316, Jeju Island, Korea	Present study	MG878635	Present study	MG878758
CEL-JJ03-03	*Pyrgomina oulastrea*	Site 316, Jeju Island, Korea	Present study	MG878636	Present study	MG878759
CEL-JJ03-04	*Pyrgomina oulastrea*	Site 316, Jeju Island, Korea	Present study	MG878637	Present study	MG878760
CEL-JJ04-02	*Pyrgomina oulastrea*	Site 316, Jeju Island, Korea	Present study	MG878638	Present study	MG878761
CEL-JJ04-03	*Pyrgomina oulastrea*	Site 316, Jeju Island, Korea	Present study	MG878639	Present study	MG878762
CEL-JJ04-04	*Pyrgomina oulastrea*	Site 316, Jeju Island, Korea	Present study	MG878640	Present study	MG878763

CEL-JJ05-02	*Pyrgomina oulastrea*	Site 316, Jeju Island, Korea	Present study	MG878641	Present study	MG878764
CEL-JJ05-03	*Pyrgomina oulastrea*	Site 316, Jeju Island, Korea	Present study	MG878642	Present study	MG878765
CEL-JJ05-04	*Pyrgomina oulastrea*	Site 316, Jeju Island, Korea	Present study	MG878643	Present study	MG878766
CEL-JJ06-02	*Pyrgomina oulastrea*	Site 316, Jeju Island, Korea	Present study	MG878644	Present study	MG878767
CEL-JJ06-03	*Pyrgomina oulastrea*	Site 316, Jeju Island, Korea	Present study	MG878645	Present study	MG878768
CEL-JJ06-04	*Pyrgomina oulastrea*	Site 316, Jeju Island, Korea	Present study	MG878646	Present study	MG878769

CEL-JJ10-02	*Pyrgomina oulastrea*	Site 316, Jeju Island, Korea	Present study	MG878647	Present study	MG878773
CEL-JJ10-03	*Pyrgomina oulastrea*	Site 316, Jeju Island, Korea	Present study	MG878648	Present study	MG878774
CEL-JJ10-04	*Pyrgomina oulastrea*	Site 316, Jeju Island, Korea	Present study	MG878649	Present study	MG878775
CEL-JJ11-02	*Pyrgomina oulastrea*	Site 316, Jeju Island, Korea	Present study	MG878661	Present study	MG878776
CEL-JJ11-03	*Pyrgomina oulastrea*	Site 316, Jeju Island, Korea	Present study	MG878662	Present study	MG878777
CEL-JJ11-04	*Pyrgomina oulastrea*	Site 316, Jeju Island, Korea	Present study	MG878663	Present study	MG878778

CEL-JJ21-02	*Pyrgomina oulastrea*	Big Rock, 1st Dive, Jeju Island, Korea	Present study	MG878650	Present study	MG878803
CEL-JJ21-03	*Pyrgomina oulastrea*	Big Rock, 1st Dive, Jeju Island, Korea	Present study	MG878651	Present study	MG878804
CEL-JJ21-04	*Pyrgomina oulastrea*	Big Rock, 1st Dive, Jeju Island, Korea	Present study	MG878652	Present study	MG878805
CEL-JJ40-01	*Pyrgomina oulastrea*	Site 323, Jeju Island, Korea	Present study	MG878653	Present study	MG878834
CEL-JJ40-02	*Pyrgomina oulastrea*	Site 323, Jeju Island, Korea	Present study	MG878654	Present study	MG878835
CEL-JJ42-02	*Pyrgomina oulastrea*	1st Dive, Beom Sum, Jeju Island, Korea	Present study	MG878655	Present study	MG878839

CEL-JJ42-03	*Pyrgomina oulastrea*	1st Dive, Beom Sum, Jeju Island, Korea	Present study	MG878656	Present study	MG878840
CEL-JJ42-04	*Pyrgomina oulastrea*	1st Dive, Beom Sum, Jeju Island, Korea	Present study	MG878657	Present study	MG878841
CEL-JJ44-03	*Pyrgomina oulastrea*	Site 324, Beom Sum, Jeju Island, Korea	Present study	MG878658	Present study	MG878845
CEL-JJ44-04	*Pyrgomina oulastrea*	Site 324, Beom Sum, Jeju Island, Korea	Present study	MG878659	Present study	MG878846
CEL-JJ44-05	*Pyrgomina oulastrea*	Site 324, Beom Sum, Jeju Island, Korea	Present study	MG878660	Present study	MG878847
AAM83	*Amphibalanus amphitrite* (Out group)	Guangdong, Xuwen, China	Yuan, S. et al.	JQ035515	Yuan, S. et al.	JQ035486

2-12-1	*Amphibalanus amphitrite* (Out group)	Tongyeong, Gyeongsangnamdo, Korea	Present study	MG878628	Present study	MG878751
